# Cu–Bpin-mediated dimerization of 4,4-dichloro-2-butenoic acid derivatives enables the synthesis of densely functionalized cyclopropanes

**DOI:** 10.3762/bjoc.21.71

**Published:** 2025-05-05

**Authors:** Patricia Gómez-Roibás, Andrea Chaves-Pouso, Martín Fañanás-Mastral

**Affiliations:** 1 Centro Singular de Investigación en Química Biolóxica e Materiais Moleculares (CiQUS), Universidade de Santiago de Compostela, 15782 Santiago de Compostela, Spain

**Keywords:** chlorocyclopropanes, copper, cyclization, 4,4-dichloro-2-butenoic acid derivatives, dimerization

## Abstract

4,4-Dichloro-2-butenoic acid derivatives are shown to undergo a rare dimerization process when reacted with bis(pinacolato)diboron under copper catalysis. The reaction provides densely functionalized products with excellent levels of chemo-, regio-, and diastereoselectivity. This high degree of functionalization makes these products versatile building blocks for the stereoselective synthesis of chlorocyclopropanes.

## Introduction

**Scheme 1:**
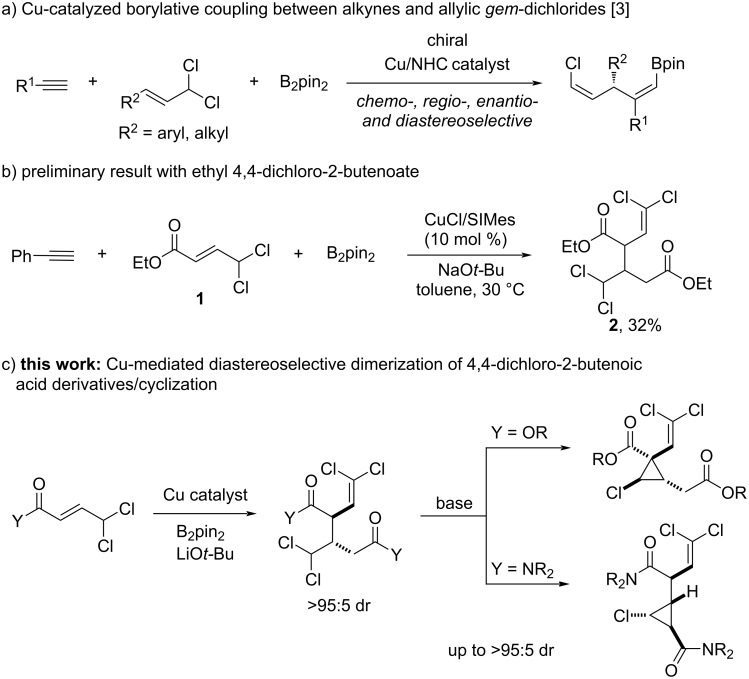
Chemodivergent reactivity observed in copper-catalyzed borylative couplings of allylic *gem*-dichlorides.

In the last years our group has been focused on the development of catalytic methodologies for the carboboration of unsaturated hydrocarbons [1–7]. In the course of our investigation of the copper-catalyzed borylative coupling of alkynes with allylic *gem*-dichlorides [3], we observed that alkyl 4,4-dichloro-2-butenoates deviated from the general reactivity trend. While allylic *gem*-dichlorides bearing aromatic and aliphatic substituents efficiently provided the allylboration product (Scheme 1a), the use of ester derivative **1** under same reaction conditions led to the formation of an unexpected product arising from the coupling of two dichloride molecules with no alkyne incorporation (Scheme 1b). We have studied this reaction and now report a catalytic methodology for the diastereoselective synthesis of these dimeric structures. The high degree of functionalization present in these molecules, which feature two ester groups, an aliphatic *gem*-dichloride and a dichloroalkene unit, offers ample opportunities for further functionalization. This is illustrated by their chemo- and diastereoselective conversion into densely functionalized cyclopropanes (Scheme 1c).

## Results and Discussion

Based on our preliminary result, we started our study by exploring the reaction between ethyl 4,4-dichloro-2-butenoate (**1**) and B_2_pin_2_ (Table 1). By using NaO*t*-Bu as base and toluene as solvent, the Cu/SIMes catalyst provided compound **2** as single reaction product, albeit in low yield and with low diastereoselectivity (Table 1, entry 1). Lowering the amount of B_2_pin_2_ to 1 equivalent was found to be beneficial (Table 1, entry 2), although the use of sub-stoichiometric amounts led to a significant decrease in reaction yield (Table 1, entry 3). We also tried to reduce the amount of base, but this caused a drop in the reaction efficiency (Table 1, entry 4). Evaluation of different bases demonstrated the important role of the base metal cation. Gratifyingly, we observed that the use of LiO*t*-Bu led to the formation of product **2** as a single diastereomer (Table 1, entry 5). A slightly lower diastereoselectivity was observed when KO*t*-Bu was used, which also gave rise to **2** in diminished yield (Table 1, entry 6). The nature of the solvent also played a role in the reaction outcome. A decrease both in efficiency and diastereoselectivity was observed when THF was used (Table 1, entry 7). The use of dichloromethane eroded the diastereoselectivity and also the chemoselectivity as shown with the additional formation of **3** as a mixture of *Z*:*E* isomers (Table 1, entry 8). Having identified the proper combination of base and solvent, we then screened different copper catalysts. Different NHCs, bisphosphines and phosphines were tested (Table 1, entries 9–14) and excellent chemo- and diastereoselectivity was observed in all cases, with SIPr providing the best result (Table 1, entry 11). Under these optimized conditions, product **2** was isolated in 60% yield as a single diastereomer. The relative configuration of **2** was determined by two-dimensional NMR analysis (see Supporting Information File 1 for details).

**Table 1:** Optimization studies.

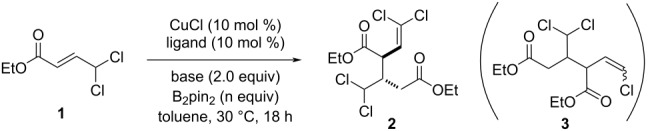					

Entry^a^	Base	B_2_pin_2_ (equiv)	Ligand	Yield **2**^b^	**2** dr^c^

1	NaO*t*-Bu	2.0	SIMes	34	73:27
2	NaO*t*-Bu	1.0	SIMes	56	70:30
3	NaO*t*-Bu	0.5	SIMes	8	n.d.
4	NaO*t-*Bu^d^	1.0	SIMes	22	72:28
5	LiO*t-*Bu	1.0	SIMes	49	>95:5
6	KO*t-*Bu	1.0	SIMes	27	92:8
7^e^	LiO*t-*Bu	1.0	SIMes	20	93:7
8^f^	LiO*t-*Bu	1.0	SIMes	41^g^	92:8
9	LiO*t-*Bu	1.0	IMes	35	>95:5
10	LiO*t-*Bu	1.0	IPr	50	>95:5
11	LiO*t-*Bu	1.0	SIPr	60	>95:5
12	LiO*t-*Bu	1.0	DPEphos	57	>95:5
13	LiO*t-*Bu	1.0	Xantphos	39	>95:5
14	LiO*t-*Bu	1.0	PCy_3_	51	>95:5

^a^Reactions run on a 0.2 mmol scale. ^b^Yield of isolated product. ^c^Diastereomeric ratio determined by GC analysis of reaction crude (structure of major diastereomer shown). ^d^1.0 equiv of NaO*t-*Bu. ^e^THF used as solvent. ^f^CH_2_Cl_2_ used as solvent. ^g^Product **3** was also obtained in 15% yield as a 1:1 mixture of *Z*:*E* isomers.

**Scheme 2:**
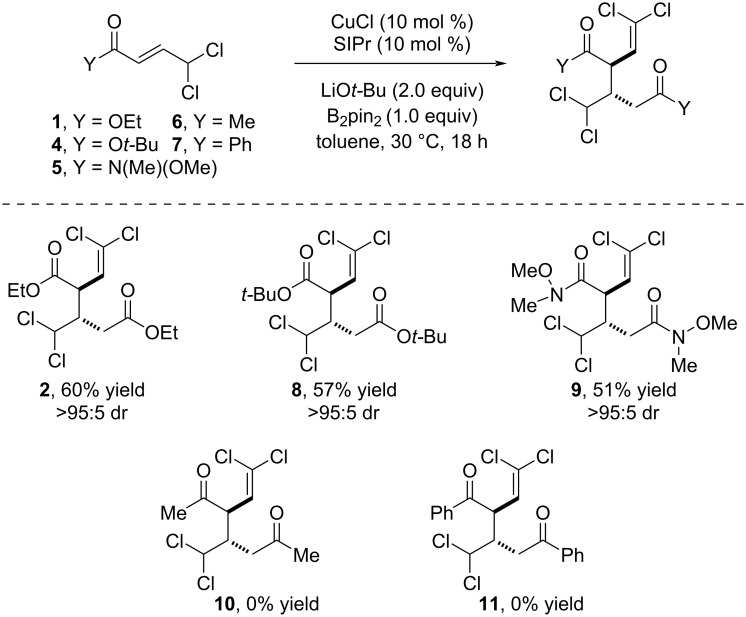
Cu-Bpin-mediated dimerization of 4,4-dichoro-2-butenoic acid derivatives.

This transformation could be efficiently applied to the dimerization of other 4,4-dichloro-2-butenoates and 4,4-dichloro-2-butenamides. The corresponding products **8** and **9** were obtained in good yield and excellent diastereoselectivity (Scheme 2). In sharp contrast, the use of *gem*-dichlorides bearing a ketone group did not result in the formation of the dimerization product and complex mixtures of products were observed in these cases.

**Scheme 3:**
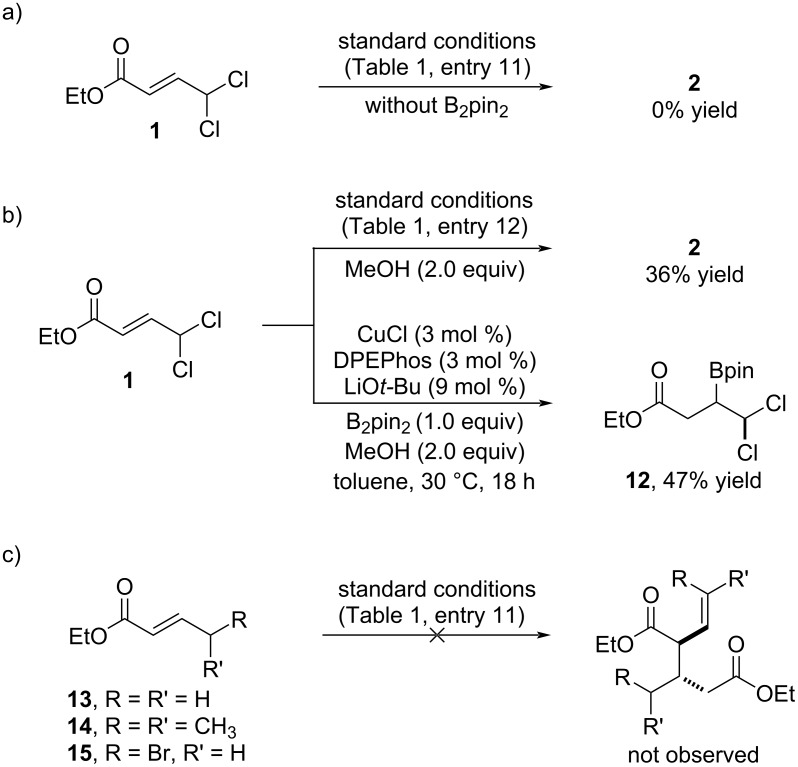
Control experiments.

To gather insight into the reaction mechanism, several control experiments were performed (Scheme 3).

We first observed that the reaction does not take place in the absence of B_2_pin_2_ (Scheme 3a). Based on the well accepted metathesis reaction of Cu(I) alkoxides with B_2_pin_2_ and the reactivity of the resulting Cu–Bpin complex towards α,β-unsaturated esters and hydrocarbons [8–15], we hypothesized that the first step of the reaction may deal with the insertion of the copper–boron bond into **1**. The dual functionality of this substrate imposed a question related to the regioselectivity of the Cu–Bpin insertion since it can potentially behave as an α,β-unsaturated ester or an allylic substrate [16–19]. To shed some light into this issue, we ran the reaction in the presence of MeOH in order to trap the potential copper intermediate by protonation. When 2 equiv of MeOH were used, we still obtained the dimerization product **2**. Nevertheless, when a catalytic amount of base was used, we only observed the formation of β-borylation product **12** (Scheme 3b). This result suggests that Cu–Bpin insertion into **1** generates a copper enolate which may engage in further steps for the formation of the dimerization product. The presence of the two chlorine atoms was found to be key for the outcome of the reaction. No dimerization product was observed when the reaction was carried out under standard conditions with ethyl crotonate derivatives bearing a methyl group, hydrogen or bromine atoms at the γ position. Either β-borylation or decomposition products were obtained in those cases (Scheme 3c).

**Scheme 4:**
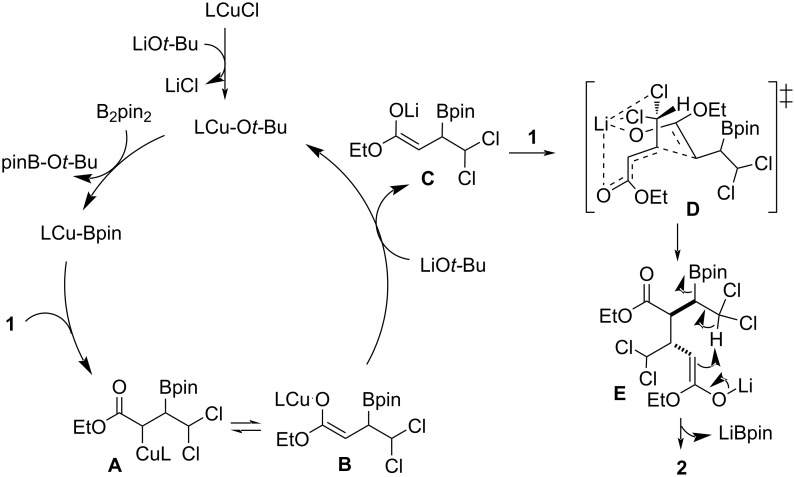
Proposed mechanism for the Cu-catalyzed dimerization of 4,4-dichoro-2-butenoic acid derivatives.

On the basis of our experimental results, we propose the following mechanism for the copper-catalyzed diastereoselective dimerization of 4,4-dichoro-2-butenoic acid derivatives (Scheme 4). Initially, the LCu–pin complex generated through reaction between LCu–O*t*-Bu and B_2_pin_2_ undergoes coordination and regioselective insertion into **1** giving rise to β-borylated organocopper species **A** which is in equilibrium with the Cu–O enolate **B** [11]. In the presence of excess of LiO*t*-Bu, a salt metathesis reaction between this base and intermediate **B** generates lithium enolate **C** and LCuO*t*-Bu to close the copper catalytic cycle. The formation of a lithium enolate is consistent with the different diastereoselectivity observed when other bases featuring different metal cations were used (Table 1, entry 2 vs entry 5), and the absence of any significant stereochemical influence from the copper complex (Table 1, entries 9–14). Lithium enolate **C** would then undergo a diastereoselective conjugate addition to a second molecule of **1**. Given the negative results observed for other crotonate derivatives (Scheme 3c), coordination between the Li cation and the two chlorine atoms via proposed transition state **D** may be crucial not only for diastereoselective control but also for the viability of this step. Finally, the new enolate **E** evolves through intramolecular proton abstraction and elimination of boryllithium [20–21]. The formation of side product **3** observed when dichloromethane was used as a solvent could be explained by protonation of intermediate **A**, followed by transmetalation of the resulting organoboron compound with CuO*t*-Bu and subsequent S_N_2’-selective allylic alkylation of **1**.

The densely functionalized structure of these dimerization products offers a versatile synthetic handle for further chemoselective functionalization. Considering the presence of two enolizable esters together with the aliphatic *gem*-dichloride, we explored the feasibility of a base-mediated formation of chlorocyclopropanes (Table 2).

**Table 2:** Synthesis of densely functionalized (2,2-dichlorovinyl)cyclopropanes by base-promoted intramolecular cyclization.

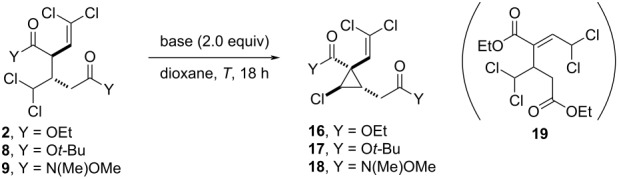					

Entry^a^	Y	Base	*T* (°C)	Product, yield (%)^b^	dr^c^

1	OEt	CsF	70	**16**, 80	50:50
2	OEt	Cs_2_CO_3_	70	**16**, 55	61:39
3	OEt	TBAF	70	**16**, 32^d^	80:20
4	OEt	TBAF	50	**16**, 70	83:17
5	O*t*-Bu	TBAF	50	**17**, 61	81:19
6	N(Me)(OMe)	TBAF	50	**18**, –	–

^a^Reactions run on a 0.1 mmol scale. ^b^Yield of isolated product. ^c^Diastereomeric ratio determined by ^1^H NMR analysis of reaction crude. ^d^Product **19** was also obtained in 23% yield.

Evaluation of bases such as metal *tert*-butoxides, phosphates, acetates, and organic amines resulted in either low conversion or decomposition of **2** (see Supporting Information File 1 for details). In contrast, the use of CsF in dioxane at 70 °C proved to be efficient and selectively provided cyclopropane **9** in good yield, albeit with no diastereoselectivity (Table 2, entry 1). Cs_2_CO_3_ was also selective for this cyclization and provided a slight increase in diastereoselectivity, although still far from satisfactory (Table 2, entry 2). A major improvement was observed when TBAF was used as base. At 70 °C, product **16** was obtained with good diastereoselectivity (80:20 dr), although in low yield mainly due to the formation of side product **19**. However, **16** could be obtained as a single product in 70% yield with 83:17 dr by decreasing the reaction temperature to 50 °C (Table 2, entry 4). Under the same optimal conditions, compound **8** could be transformed into cyclopropane **17** in 61% yield with 81:19 dr (Table 2, entry 5). It is important to note that (2,2-dichlorovinyl)cyclopropanes represent an important class of compounds present in a range of bioactive compounds such as permethrin or alpha-cypermethrin, which are commonly used as insecticides [22–23]. The present transformation provides access to densely functionalized (2,2-dichlorovinyl)cyclopropanes, thus representing a potential platform for the synthetic diversification on these important scaffolds.

**Scheme 5:**
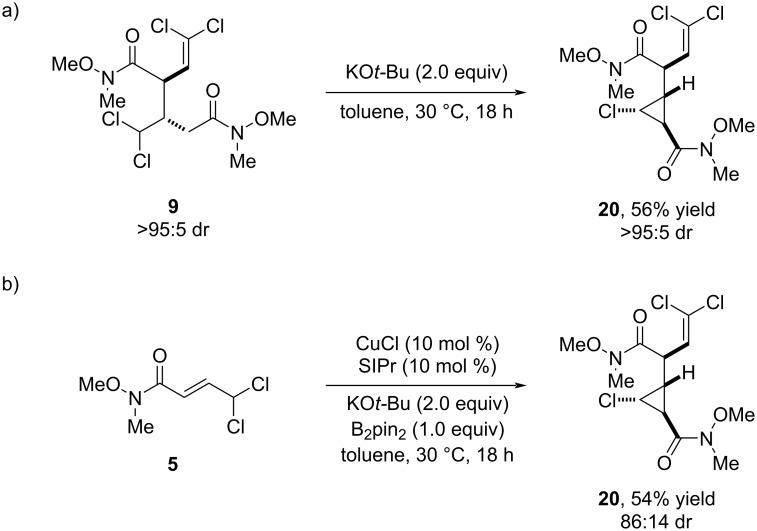
a) KO*t*-Bu-mediated intramolecular cyclization of **9**. b) Direct formation of cyclopropane **20** from *gem*-dichloride **5** using KO*t*-Bu as base.

Surprisingly, when the TBAF-mediated cyclization was attempted on bisamide **9** the corresponding cyclopropane **18** was not formed (Table 2, entry 6). The use of other mild bases at different temperatures also resulted unproductive. However, when the reaction was carried out with KO*t*-Bu, we observed the selective conversion of **9** into product **20** featuring a different cyclopropane scaffold. Slight modification of the reaction conditions allowed us to obtain product **20** in 56% yield as a single diastereomer (Scheme 5a). Taking advantage of the twofold utility of KO*t*-Bu, we explored the formation of this new cyclopropane structure directly from *gem*-dichloride **5**. By using this base under standard conditions, product **20** was selectively obtained in similar yield, albeit with diminished diastereoselectivity (Scheme 5b).

## Conclusion

In summary, we have discovered an unanticipated Cu–Bpin-promoted diastereoselective dimerization of 4,4-dichloro-2-butenoic acid derivatives. The reaction occurs via initial Cu–Bpin insertion followed by keto–enol isomerization and salt metathesis to generate a lithium enolate which is then trapped by a second molecule of the 4,4-dichloro-2-butenoic acid derivative. We have observed that the use of lithium as base metal cation is key to achieve excellent levels of diastereoselectivity. Our study also highlights, how the dimerization products can be selectively converted into different densely functionalized cyclopropane scaffolds depending on the nature of the carboxylic acid derivative.

## Supporting Information

File 1 Experimental procedures, characterization data and copies of NMR spectra.

## Data Availability

All data that supports the findings of this study is available in the published article and/or the supporting information of this article.
