# Supplementary material for: Cu–Bpin-mediated dimerization of 4,4-dichloro-2-butenoic acid derivatives enables the synthesis of densely functionalized cyclopropanes
Source: Beilstein J Org Chem. 2025 May 5;21:877–83. doi: 10.3762/bjoc.21.71 (PMC12067098; doi:10.3762/bjoc.21.71)
Supplement: File 1 — Experimental procedures, characterization data and copies of NMR spectra. [file Beilstein_J_Org_Chem-21-877-s001.pdf]

BEILSTEIN JOURNAL OF ORGANIC CHEMISTRY

Supporting Information

for

Cu–Bpin-mediated dimerization of 4,4-dichloro-2-butenoic acid derivatives enables the synthesis of densely functionalized cyclopropanes

Patricia Gómez-Roibás, Andrea Chaves-Pouso and Martín Fañanás-Mastral

Beilstein J. Org. Chem. **2025,** *21,* 877–883. doi:10.3762/bjoc.21.71

Experimental procedures, characterization data and copies of NMR spectra

License and Terms: This is a supporting information file under the terms of the Creative Commons Attribution License (https://creativecommons.org/licenses/by/4.0). Please note that the reuse, redistribution and reproduction in particular requires that the author(s) and source are credited and that individual graphics may be subject to special legal provisions.
The license is subject to the *Beilstein Journal of Organic Chemistry* terms and conditions: (https://www.beilstein-journals.org/bjoc/terms)

Table of contents

1. General methods S2

2. List of starting materials and ligands... S3

3. Synthesis of 4,4-dichloro-2-butenoic acid derivatives S4

4. General procedures S6

   - General procedure for the copper-catalyzed dimerization of 4,4-dichloro-2-butenoic acid derivatives (general procedure B).. S6
   - General procedure for the synthesis of (2,2-dichlorvinyl)cyclopropanes (general procedure C) S6
   - Procedures for the synthesis of chlorocyclopropane **20**. S6

5. Experimental details for Scheme 3 .... S7

6. Optimization studies towards the synthesis of cyclopropane **16** S8

7. Product characterization S9

8. NMR spectra S12

9. Stereochemical determination of products by NOESY experiment.................... S27

1. General methods

- All reactions were performed under argon atmosphere using oven-dried glassware and using standard Schlenk techniques. Solvents were dried using an MBraun SPS 800 system. All chemicals were purchased from Aldrich Chemical Co. Ltd., Alfa Aesar, Fluorochem Ltd. or TCI Europe N.V. chemical companies and used without further purification, unless otherwise noted.

- Analytical thin-layer chromatography was carried out on silica-coated aluminium plates (silica gel 60 F254 Merck) and components were visualized by UV light and $KMnO_4$ staining. Flash column chromatography was performed on silica gel 60 (Merck, 230–400 mesh) without previous deactivation.

- GC–MS analyses were performed in an Agilent instrument GC-6890N equipped with Chemical Ionization (CI) MS-5973 detector.

- High resolution mass spectrometry was carried out on a Bruker micro TOF spectrometer using APCI.

- ^{1}H, ^{13}C, COSY, HSQC, HMBC and NOESY NMR experiments were carried out using a Varian Inova 500 MHz or a Varian Mercury 300 MHz NMR spectrometer. Chemical shift values are reported in ppm with the solvent resonance as the internal standard ($CHCl_3$: δ 7.26 for ^{1}H, δ 77.2 for ^{13}C). Coupling constants (*J*) are given in hertz (Hz). Multiplicities are reported as follows: s = singlet, d = doublet, t = triplet, q = quartet, m = multiplet or as a combination of them.

- In order to preclude side reactions, B_2pin_2 was dried over Na_2SO_4 prior to being used.

2. List of starting materials and ligands.

Substrates **1** and **4–7** were prepared according to a modified literature procedure[1] (see below, general procedure A). Substrates **13–15** and ligands were purchased from commercial sources and used without further purification.

- Substrates:

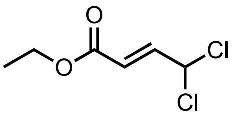

1

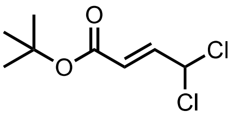

4

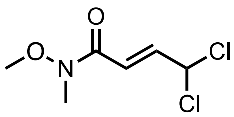

5

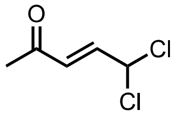

6

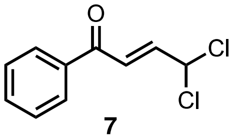

7

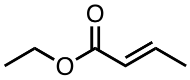

13

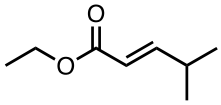

14

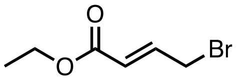

15

- Ligands:

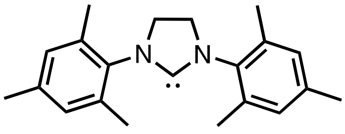

SIMes

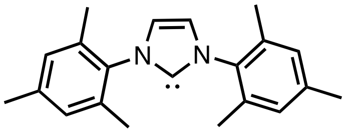

IMes

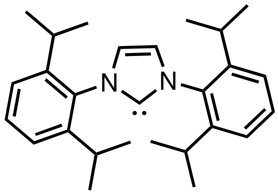

IPr

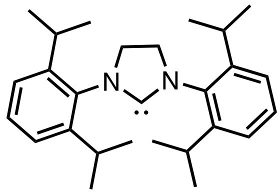

SIPr

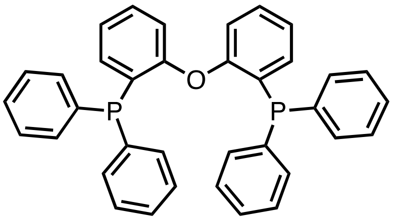

DPEphos

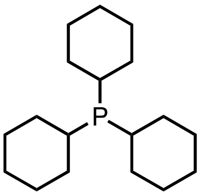

PCy_3

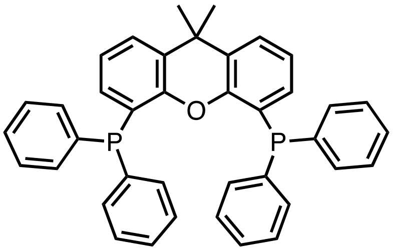

Xantphos

[1] Giannerini, M.; Fañanás-Mastral, M.; Feringa, B. L. *J. Am. Chem. Soc.* **2012**, *134*, 4108–4111.

3. Synthesis of 4,4-dichloro-2-butenoic acid derivatives

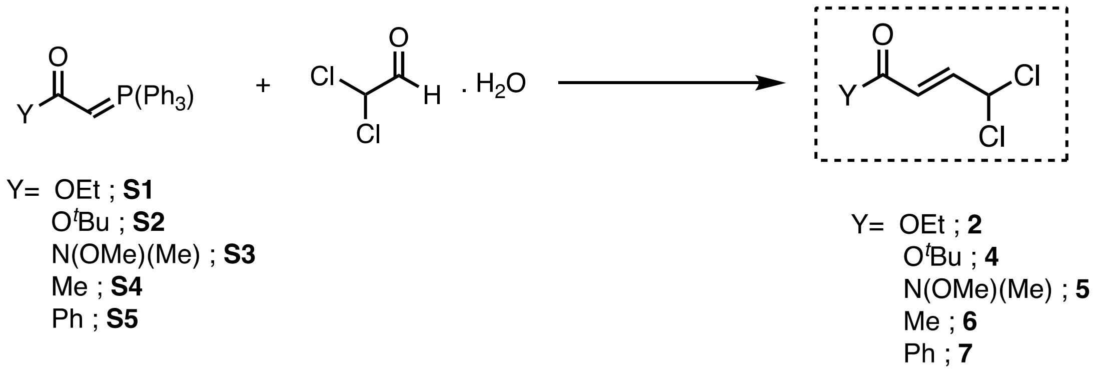

General procedure A:

In a two-necked flask under argon atmosphere equipped with a magnetic stirring bar, 1,1-dichloroacetaldehyde hydrate (1 equiv) was added to a solution of the corresponding phosphorus ylide (1.5 equiv) in dry DCM at 0 °C. The mixture was allowed to reach room temperature and stirred for 4 hours. The resulting mixture was extracted with a saturated aqueous solution of NH_4Cl and dichloromethane (3 × 30mL). The combined organic layers were dried over anhydrous Na_2SO_4, filtered, and the solvent was removed under reduced pressure. The crude product was purified through flash column chromatography using the indicated mixture of solvents as eluent.

Note: phosphorus ylides **S1**, **S2**, **S3, S4** and **S5** were purchased from commercial sources.

Ethyl (*E*)-4,4-dichlorobut-2-enoate (1)[1]

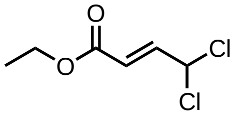

Synthesized from **S1** according to general procedure A. Yellow oil obtained in 42% yield (569.6 mg)after flash column chromatography on silica gel (hexane:AcOEt, 200:1). **^{1}H NMR** (300 MHz, $CDCl_3$**)** δ 7.02 (dd, *J* = 15.3, 6.8 Hz, 1H), 6.25 (d, *J* = 6.8 Hz, 1H), 6.15 (dd, *J* = 15.3, 1.1 Hz, 1H), 4.25 (q, *J* = 7.1 Hz, 2H), 1.32 (t, *J* = 7.1 Hz, 3H). **^{13}C NMR** (300 MHz, $CDCl_3$) δ 164.9 (C), 142.4 (CH), 123.1 (CH), 68.1 (CH), 61.2 (CH_2), 14.1 (CH_3) ppm.

***tert*-Butyl (*E*)-4,4-dichlorobut-2-enoate (4)**[1]

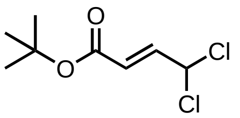

Synthesized from **S2** according to general procedure A. Yellow oil obtained in 43% yield (728.4 mg) after flash column chromatography on silica gel (hexane:AcOEt, 200:1). **^{1}H NMR** (300 MHz, $CDCl_3$) δ 6.86 (dd, *J* = 15.6, 7.3 Hz, 1H), 6.22 (d, *J* = 6.9 Hz, 1H), 6.02 (d, *J* = 15.2 Hz, 1H), 1.45 (s, 9H) ppm. **^{13}C NMR** (300 MHz, $cdcl_3$) δ 163.7 (C), 141.4 (CH), 124.9 (CH), 82.0 (C), 68.2 (CH), 27.7 (3xCH_3) ppm.

(*E*)-4,4-Dichloro-*N*-methoxy-*N*-methylbut-2-enamide (5)

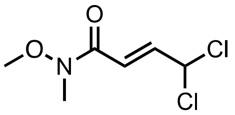

Synthesized from **S3** according to general procedure A. Colorless oil obtained in 67% yield (663.3 mg) after flash column chromatography on silica gel (hexane:AcOEt, 100:5). **^{1}H NMR** (300 MHz, $CDCl_3$) δ 7.05 (dd, *J* = 15.1, 6.8 Hz, 1H), 6.74 (dd, *J* = 15.1, 1.1 Hz, 1H), 6.32 (dd, *J* = 6.8, 1.0 Hz, 1H), 3.73 (s, 3H), 3.27 (s, 3H) ppm. **^{13}C NMR** (300 MHz, $CDCl_3$) δ 164.6 (C), 141.8 (CH), 120.7 (CH), 68.8 (CH), 62.0 (CH_3), 32.4 (CH_3) ppm. **HRMS (APCI)** Calc. for $C_6H_{10}Cl_2NO_2$ [M+H$^+$]: 198.0083, found: 198.0088.

(*E*)-5,5-Dichloropent-3-en-2-one (6)

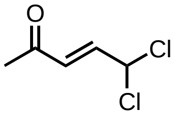

Synthesized from **S4** according to general procedure A. Yellow oil obtained in 6% yield (124.5 mg) after flash column chromatography on silica gel (hexane:AcOEt, 200:1). **^{1}H NMR** (300 MHz, $CDCl_3$) δ 6.83 (dd, *J* = 15.5, 7.0 Hz, 1H) 6.28 (m, 2H), 2.31 (s, 3H) ppm. **^{13}C NMR** (300 MHz, $CDCl_3$) δ 197.0 (C), 140.9 (CH), 130.2 (CH), 68.5 (CH), 27.9 (CH_3) ppm.

(*E*)-4,4-Dichloro-1-phenylbut-2-en-1-one (7)

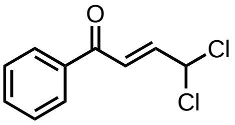

Synthesized from **S5** according to general procedure A. Yellow oil obtained in 42% yield (272.3 mg) after flash column chromatography on silica gel (hexane:AcOEt, 200:1). **^{1}H NMR** (300 MHz, $CDCl_3$) δ 7.80 (m, 2H), 7.55 (m, 3H), 7.21 (dd, *J* = 15.0, 0.9 Hz, 1H), 7.07 (dd, *J* = 15.1, 6.3 Hz, 1H), 6.40 (dd, *J* = 6.4, 0.9 Hz, 1H) ppm. **^{13}C NMR** (300 MHz, $CDCl_3$) δ 189.2(C), 141.9(CH), 136.9(C), 133.6 (2xCH), 128.89 (2xCH), 128.7 (CH), 126.1 (CH), 68.7 (CH) ppm. **HRMS (APCI)** Calc. for $C_{10}H_9Cl_2O$ [M+H$^+$]: 215.0025, found: 215.0018.

4. General procedures

- **General procedure for the copper-catalyzed dimerization of 4,4-dichloro-2-butenoic acid derivatives (general procedure B)**

A dry reaction tube equipped with a magnetic stirring bar was charged with CuCl (10 mol %, 1.98 mg) and SIPr (10 mol %, 9.56 mg) and placed under argon via three evacuation/backfill cycles. Then, dry toluene (0.5 mL) was added, and the resulting solution was stirred for 30 min. The obtained solution was added to a Schlenk flask containing B_2pin_2 (0.2 mmol, 50.79 mg) and LiO*t*-Bu (0.4 mmol, 32.02 mg) followed by vacuum/argon cycles. A solution of the corresponding 4,4-dichloro-2-butenoic acid derivative (0.2 mmol) in toluene (1 mL) was then added. The mixture was stirred at 30 °C for 18 h and the resulting crude was filtered through celite to remove the catalyst. After this, the mixture was concentrated under reduced pressured and analyzed by GC–MS and ^{1}H NMR spectroscopy to determine the conversion and product distribution. Finally, the crude product was purified through flash column chromatography using the indicated mixture of solvents as eluent.

- **General procedure for the synthesis of (2,2-dichlorvinyl)cyclopropanes (General procedure C)**

In a pre-dried vial with a magnetic stirring bar, a solution of the dimerization product was added and heated at 50 °C for 18 h under argon. The resulting crude was extracted with a saturated aqueous solution of NH_4Cl and dichloromethane (3 × 10 mL). The organic phase was dried with anhydrous Na_2SO_4, filtered, and concentrated under reduced pressure. The crude obtained was purified by column chromatography using the indicated mixture of solvents as eluent.

- **Procedures for the synthesis of chlorocyclopropane 20.**

From **5** (procedure D): A dry reaction tube equipped with a magnetic stirring bar was charged with CuCl (10 mol %, 1.98 mg) and SIPr (10 mol %, 9.56 mg) and placed under argon via three evacuation/backfill cycles. Then, dry toluene (0.5 mL) was added, and the resulting solution was stirred for 30 min. The obtained solution was added to a Schlenk flask, containing B_2pin_2 (0.2 mmol, 50.79 mg) and KO*t*-Bu (0.4 mmol, 44.88 mg) followed by vacuum/argon cycles. A solution of **5** (0.2 mmol, 39.40 mg) in toluene (1 mL) was then added. The mixture was stirred at 30 °C for 18 h. Finally, the crude product was purified through flash column chromatography (AcOEt/hexane 60:40).

From **9** (procedure E): A pre-dried vial with a magnetic stirring bar was charged with KO*t*-Bu (0.2 mmol, 22.44 mg) and placed under argon via three evacuation/backfill cycles. Then, a solution of **9** (0.1 mmol, 39.60 mg) in toluene (1 mL) was added and the resulting mixture was stirred at 30 °C for 18 h. The solution was extracted with a saturated aqueous solution of NH_4Cl and dichloromethane (3 × 10 mL). The combined organic layers were dried with anhydrous Na_2SO_4, filtered and concentrated under reduced pressure. The crude product was purified by column chromatography (AcOEt/hexane 60:40).

5. Experimental details for Scheme 3

Scheme 3a: performed according to general procedure B without the addition of B_2pin_2.

Scheme 3b:

Synthesis of **2**: performed according to general procedure B. To the final mixture MeOH (0.4 mmol, 16.2 μL) was added.

Synthesis of **12**: A dry reaction tube equipped with a magnetic stirring bar was charged with CuCl (3 mol %, 0.6 mg) and DPEPhos (3 mol %, 3.2 mg) and placed under argon via three evacuation/backfill cycles. Then, dry toluene (0.5 mL) was added, and the resulting solution was stirred for 30 min. The obtained solution was added to a Schlenk flask containing B_2pin_2 (0.2 mmol, 50.8 mg) and LiO*t*-Bu (9 mol %, 1.5 mg) followed by three vacuum/argon cycles. A solution of the corresponding 4,4-dichloro-2-butenoic acid derivative (0.2 mmol) in toluene (1 mL) was then added. Finally, MeOH (0.4 mmol, 16.2 μL) was added and the mixture stirred at 30 °C for 18 h. The resulting crude was filtered through celite to remove the catalyst. After this, the mixture was concentrated under reduced pressure and analyzed by GC–MS and ^{1}H NMR spectroscopy to determine the conversion and product distribution.

6. Optimization studies towards the synthesis of cyclopropane 16

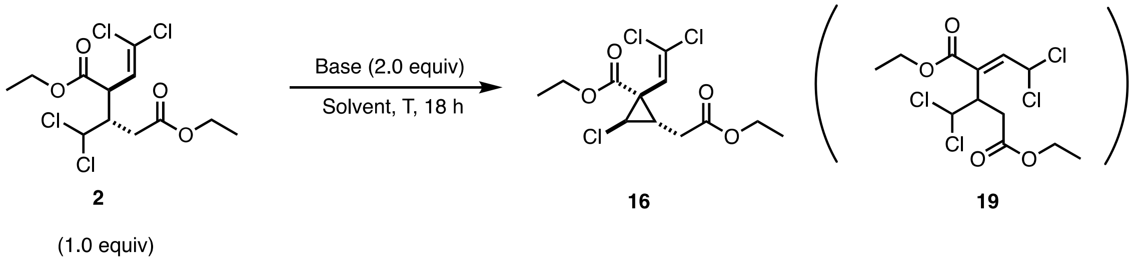

Entry[a]	Base	T (°C)	Solvent	Conv. [b]	Yield **16** (%)[c]	dr[d]
**1**	DBU	40	DCM	100	-	-
**2**	DBU	0	DCM	100	-	-
**3**	KO*t*Bu	rt	THF	100	-	-
**4**	KF	100	Dioxane	4	-	-
**5**	CsF	100	Dioxane	100	65	54:46
**6**	CsF	70	Dioxane	100	80	50:50
**7**	CsF	50	Dioxane	100	-	-
**8**	Na_2CO_3	70	Dioxane	17	-	-
**9**	K_2CO_3	70	Dioxane	88	-	-
**10**	Cs_2CO_3	70	Dioxane	100	55	61:39
**11**	Na_3PO_4	70	Dioxane	17	-	-
**12**	NaO*t*Bu	70	Dioxane	100	-	-
**13**	Et_3N or DIPEA	70	Dioxane	<10	-	-
**14**	KOAc	70	Dioxane	42	-	-
**15**[e]	TBAF	70	Dioxane	100	32	80:20
**16**	TBAF	50	Dioxane	100	70	83:17
**17**[f]	TBAF	50	Toluene	100	35	89:11
**18**	TBAF	50	THF	100	-	-

[a] Reactions run on a 0.1 mmol scale and all yield values refer to isolated products. [b] Conversion was determined by ^{1}H NMR. [c] Yield of isolated product [d]Diastereomeric ratio determined by ^{1}H NMR of reaction crude [e] Product **19** was observed (yield: 23%, dr; >99:1) [f] Product **19** was observed (yield: 20%, dr; >99:1).

7. Product characterization

Diethyl (2*R,3*S**)-3-(dichloromethyl)-2-(2,2-dichlorovinyl)pentanedioate (2)**

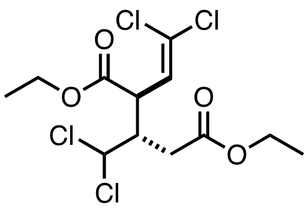

Synthesized from **1** according to general procedure B. Yellow oil obtained in 60% yield with >95:5 dr after column chromatography (DCM/hexane, 80:20).
^{1}H NMR (500 MHz, $CDCl_3$) δ 6.02 (d, *J* = 3.0 Hz, 1H), 5.87 (d, *J* = 10.6 Hz, 1H), 4.24 – 4.15 (m, 4H), 3.75 (dd, *J* = 10.5, 8.9 Hz, 1H), 3.26 (dddd, *J* = 8.9, 7.7, 4.1, 2.9 Hz, 1H), 2.85 (dd, *J* = 17.2, 4.1 Hz, 1H), 2.46 (dd, *J* = 17.2, 7.7 Hz, 1H), 1.31 (m, 6H) ppm. **^{13}C NMR** (500 MHz, $CDCl_3$) δ 171.4 (C), 169.9 (C), 125.9(C), 124.2 (CH), 74.4 (CH), 61.9 (CH_2), 61.2 (CH_2), 48.6 (CH), 46.7 (CH), 32.3 (CH_2), 14.1 (CH_3), 14.0 (CH_3) ppm. **HRMS (APCI)** Calc. for $C_{12}H_{16}Cl_4O_4$ [M+H$^+$]: 364.9875, found: 364.9874.

Diethyl 2-(2-chlorovinyl)-3-(dichloromethyl)pentanedioate (3-*Z* and 3-*E*)

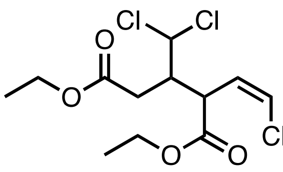

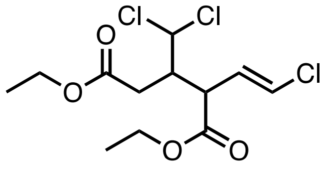

Obtained as a side product in the synthesis of **2** when reaction was carried out using DCM as solvent. Yellow oil obtained in 15% yield as a 1:1 mixture of *Z/E* isomers after column chromatography (DCM/hexane, 75:25).

^{1}H NMR (300 MHz, $CDCl_3$) δ 6.21 (d, *J* = 7.3 Hz, 1H **3-*Z***), 6.17 (d, *J* = 13.2 Hz, 1H **3-*E***), 5.96 (d, *J* = 3.0 Hz, , 1H **3-*Z***), 5.88 (d, *J* = 3.3 Hz, 1H **7-*E***), 5.80 – 5.70 (m, , 1H **3-*Z*** + 1H **3-*E***), 4.13 – 4.08 (m, , 4H **3-*Z*** + 4H **3-*E***), 3.91 (dd, *J* = 9.6 Hz, 1H **3-*E***), 3.25 (m, 2H **3-*Z*** + 1H **3-*E***), 2.78 – 2.72 (m, 1H **3-*Z*** + 1H **3-*E***), 2.42 – 2.32 (m, 1H **3-*Z*** + 1H **7-*E***), 1.24 – 1.19 (m, 6H **3-*Z*** + 6H **3-*E***) ppm. **^{13}C NMR** (500 MHz, $CDCl_3$) δ 170.7 (C **3-*Z***), 170.3 (C **3-*E***), 169.8 (C **3-*Z***), 169.6 (C **3-*E***), 126.7 (CH **3-*Z***), 125.2 (CH **3-*E***), 122.2 (CH **3-*Z***), 122.1 (CH **3-*E***), 73.7 (CH **3-*Z***), 73.6 (CH **3-*E***), 60.8 (CH_2 **3-*Z***), 60.7 (CH_2 **3-*E***), 60.2 (CH_2 **3-*Z***), 60.0 (CH_2 **3-*E***), 49.2 (CH **3-*Z***), 45.6 (CH **3-*E***), 45.4 (CH **3-*Z***), 45.1 (CH **3-*E***), 31.7 (CH_2 **3-*Z***), 31.3 (CH_2 **3-*E***), 13.0 (2xCH_3 **3-*Z* +** 2xCH_3 **3-*E***) ppm. **HRMS (APCI)** Calc. for $C_{12}H_{17}Cl_3O_4$ [M+H$^+$]:: 331.0265, found: 331.0267.

Di-*tert*-butyl (2*R,3*S**)-3-(dichloromethyl)-2-(2,2-dichlorovinyl)pentanedioate (8)**

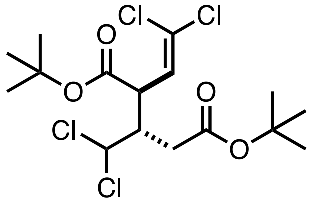

Synthesized from **4** according to general procedure B. Yellow oil obtained in 57% yield with >95:5 dr after column chromatography (DCM/hexane, 80:20).

^{1}H NRM (300 MHz, $CDCl_3$) δ 6.03 (d, *J* = 2.7 Hz, 1H), 5.88 (d, *J* = 10.4 Hz, 1H), 3.64 (dd, *J* = 10.7, 8.7 Hz, 1H), 3.26 – 3.14 (m, 1H), 2.77 (dd, *J* = 16.7, 3.6 Hz, 1H), 2.35 (dd, *J* = 17.4, 7.0 Hz, 1H), 1.49 (s, 18H) ppm. **^{13}C NMR** (300 MHz, $CDCl_3$) δ 170.6 (C), 169.1 (C), 125.0 (C), 124.7 (CH), 82.8 (C), 81.5 (C), 74.7 (CH), 49.9 (CH), 46.5 (CH), 33.5 (CH_2), 28.1 (3xCH_3), 27.9 (3xCH_3) ppm. **HRMS (APCI)** Calc. for $C_{16}H_{24}Cl_4O_4$ [M+H$^+$]: 421. 0501, found: 421.0506.

(2*R,3*S**)-3-(Dichloromethyl)-2-(2,2-dichlorovinyl)-*N*1,*N*5-dimethoxy-*N*1,*N*5-dimethylpentanediamide (9)**

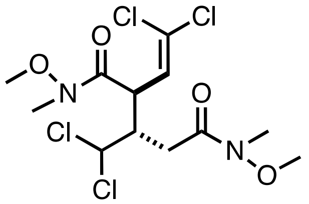

Synthesized from **5** according to general procedure B. Yellow oil obtained in 51% yield with >95:5 dr after column chromatography (AcOEt/hexane, 60:40).

^{1}H NMR (300 MHz, $CDCl_3$) δ 5.96 (d, *J* = 2.9 Hz, 1H), 5.80 (d, *J* = 10.7 Hz, 1H), 4.11 (t_{ap}, *J* = 10.2 Hz, 1H), 3.68 (s, 6H), 3.50 (dd, *J* = 8.1, 3.7 Hz, 1H), 3.15 (s, 6H), 2.81 (m, 1H), 2.67 (dd, *J* = 17.2, 7.1 Hz, 1H) ppm. **^{13}C NMR** (300MHz, $CDCl_3$) δ 171.6 (C), 169.9 (C), 125.8 (CH), 124.2 (C), 75.6 (CH), 61.6 (CH_3), 61.3 (CH_3), 45.8 (CH), 45.1 (CH), 32.4 (CH_3), 30.9 (CH_3), 29.3 (CH_2) ppm. **HRMS (APCI)** Calc. for $C_{12}H_{19}Cl_4N_2O_4$ [M+H$^+$]: 395.0093, found: 329.0094.

Ethyl (1*R,2*R**,3*S**)-2-chloro-1-(2,2-dichlorovinyl)-3-(2-ethoxy-2-oxoethyl) cyclopropane-1-carboxylate (16)**

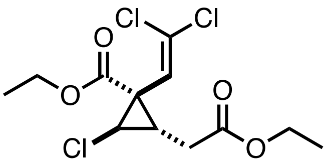

Synthesized from **2** according to general procedure C. Yellow oil obtained in 70% yield with 83:17 dr after column chromatography (DCM/hexane, 80:20).

^{1}H NRM (500 MHz, $CDCl_3$) δ 5.94 (s, 1H), 4.24 – 4.04 (m, 4H), 3.71 (d, *J* = 5.8 Hz, 1H), 2.72 (dd, *J* = 16.8, 7.8 Hz, 1H), 2.58 (dd, *J* = 16.8, 7.0 Hz, 1H), 1.82 (dd, *J* = 7.4, 5.8, 1H) Hz, 1.20 (m, 6H) ppm. **^{13}C NRM** (500 MHz, $CDCl_3$) δ 169.9 (C), 167.0 (C), 128.5 (C), 123.2 (CH), 60.1 (CH_2), 59.8 (CH_2), 42.7 (CH), 34.2 (CH), 33.0 (C), 30.3 (CH_2), 13.1 (2xCH_3) ppm. **HRMS (APCI)** Calc. for $C_{12}H_{15}Cl_3O_4$ [M+H$^+$]: 329. 0109, found: 329.0113.

***tert*-Butyl (1*R**,2*R**,3*S**)-2-(2-(*tert*-butoxy)-2-oxoethyl)-3-chloro-1-(2,2-dichlorovinyl) cyclopropane-1-carboxylate (17)**

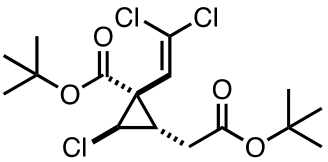

Synthesized from **8** according to general procedure B. Yellow oil obtained in 61% yield with 81:19 dr after column chromatography (DCM/hexane, 80:20).

^{1}H NMR (300 MHz, $CDCl_3$) δ 6.00 (s, 1H), 3.74 (d, *J* = 5.8 Hz, 1H), 2.69 (dd, *J* = 17.0, 7.9 Hz, 1H), 2.57 (dd, *J* = 17.0, 6.9 Hz, 1H), 1.83 (dd, 6.9 Hz, 5.8 Hz, 1H), 1.48 (s, 18H) ppm. **^{13}C NMR** (300 MHz, $CDCl_3$) δ 169.3 (C), 165.9 (C), 127.7 (C), 123.5 (CH), 81.7 (C), 80.2 (C), 42.3 (CH), 34.2 (CH), 33.8 (CH), 31.3 (CH_2), 27.1 (3xCH_3), 26.9 (3xCH_3) ppm. **HRMS (APCI)** Calc. for $C_{16}H_{23}Cl_3O_4$ [M+H$^+$-C_8H_{18}O]: 254.9372, found: 254.9377.

Diethyl (*E*)-2-(2,2-dichloroethylidene)-3-(dichloromethyl)pentanedioate (19)

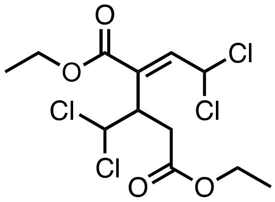

Obtained as a side product in the synthesis of **16** when reaction was carried out at 70 ºC. Yellow oil obtained in 23% yield with >99:1 dr after column chromatography (DCM/hexane, 70:30).

^{1}H NMR (300 MHz, $CDCl_3$) δ 6.99 (d, *J* = 10.0 Hz, 1H), 6.70 (d, *J* = 9.9 Hz, 1H), 6.13 (d, *J* = 9.8 Hz, 1H), 4.25 – 3.99 (m, 4H), 3.57 (td, *J* = 10.0, 3.6 Hz, 1H), 2.94 (dd, *J* = 16.4, 3.6 Hz, 1H), 2.78 (dd, *J* = 16.4, 10.0 Hz, 1H), 1.28 (m, 6H) ppm. **^{13}C NRM** (300MHz, $CDCl_3$) δ 169.9 (C), 163.8 (C), 140.9 (C), 127.7 (CH), 72.4 (CH), 64.2 (CH), 60.9 (CH_2), 60.3 (CH_2), 46.9 (CH), 34.9 (CH_2), 13.4 (2XCH_3) ppm. **HRMS (APCI)** Calc. for $C_{12}H_{16}Cl_4O_4$ [M+H$^+$]: 364.9878, found: 364.9879.

(1*R,2*S**,3*R**)-2-Chloro-3-((*R**)-4,4-dichloro-1-(methoxy(methyl)amino)-1-oxobut-3-en-2-yl)-*N*-methoxy-*N*-methylcyclopropane-1-carboxamide (20)**

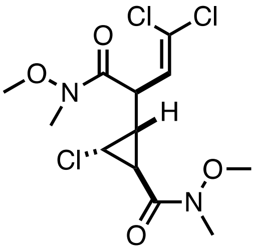

Synthesized from **5** or **9** according to either procedure D or E. Yellow oil obtained in 56% yield with >95:5 dr after column chromatography (AcOEt/hexane, 60:40).

^{1}H NMR (300 MHz, $CDCl_3$) δ 6.05 (d, *J* = 10.6 Hz, 1H), 4.04 (t_{ap}, *J* = 10.2 Hz, 1H), 3.79 (s, 3H) 3.78 (s, 3H), 3.71 (dd, *J* = 7.6, 3.5 Hz, 1H), 3.25 (s, 3H), 3.24 (s,3H), 2.45 (bs, 1H), 2.32 (ddd, *J* = 10.0, 7.6, 6.1 Hz, 1H) ppm. **^{13}C NMR** (500 MHz, $CDCl_3$) δ 169.4 (C), 169.1(C), 125.8 (C), 122.4 (CH), 74.0 (CH), 60.8 (CH_3), 60.6 (CH_3), 41.8 (CH), 38.2 (CH), 29.89 (2xCH_3), 27.58 (CH) ppm. **HRMS (APCI**) Calc. for $C_{12}H_{17}Cl_3N_2O_4$ [M+H$^+$]: 359.0332, found: 359.0336.

8. NMR spectra

1H NMR (CDCl3, 300MHz)

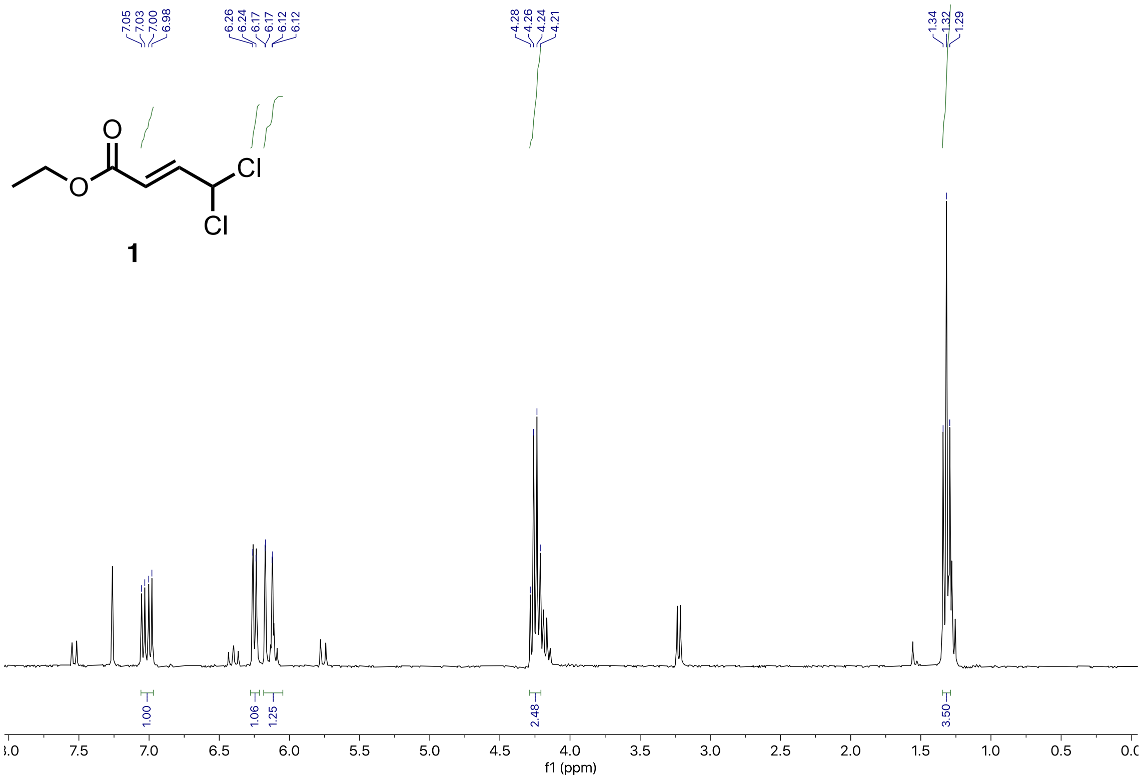

13C NMR (CDCl3, 300 MHz)

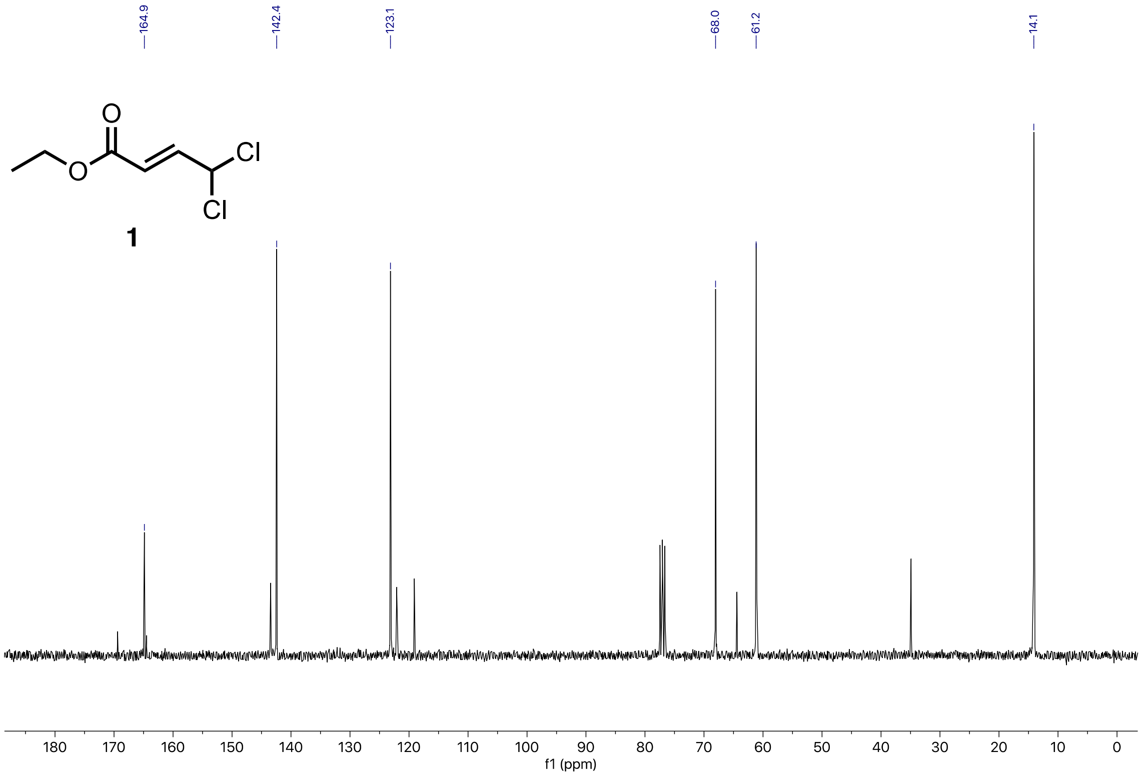

1H NMR (CDCl3, 300MHz)

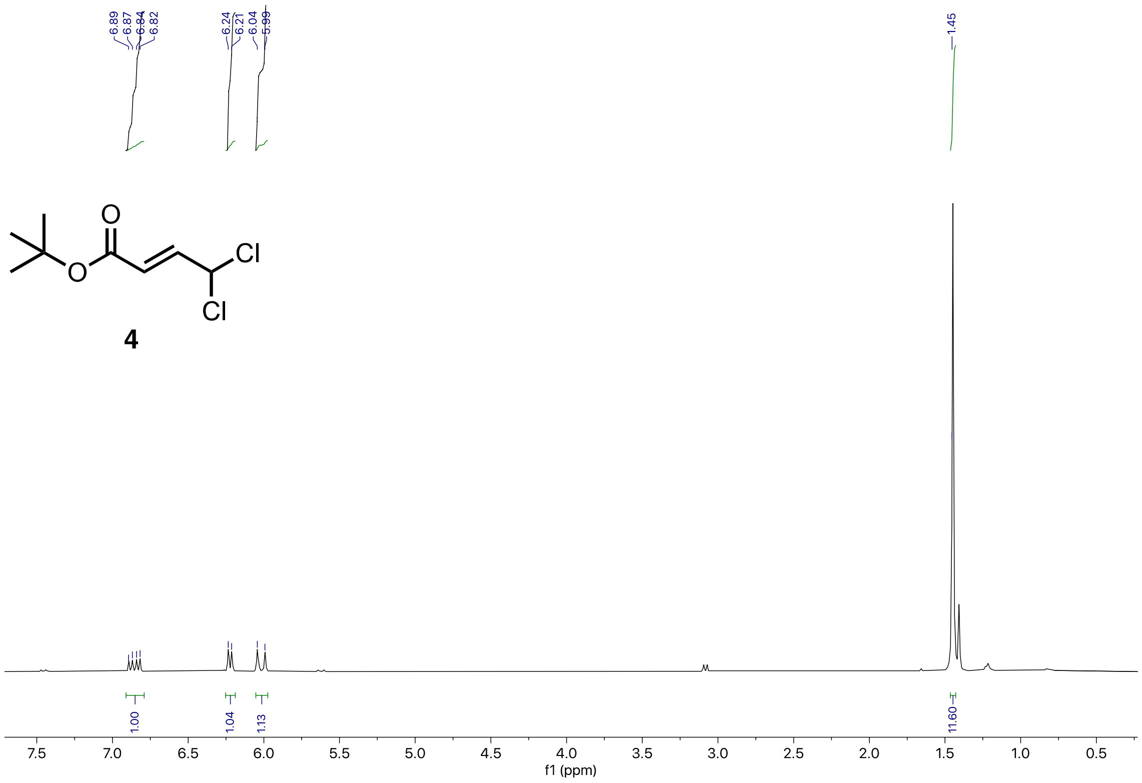

13C NMR (CDCl3, 300 MHz)

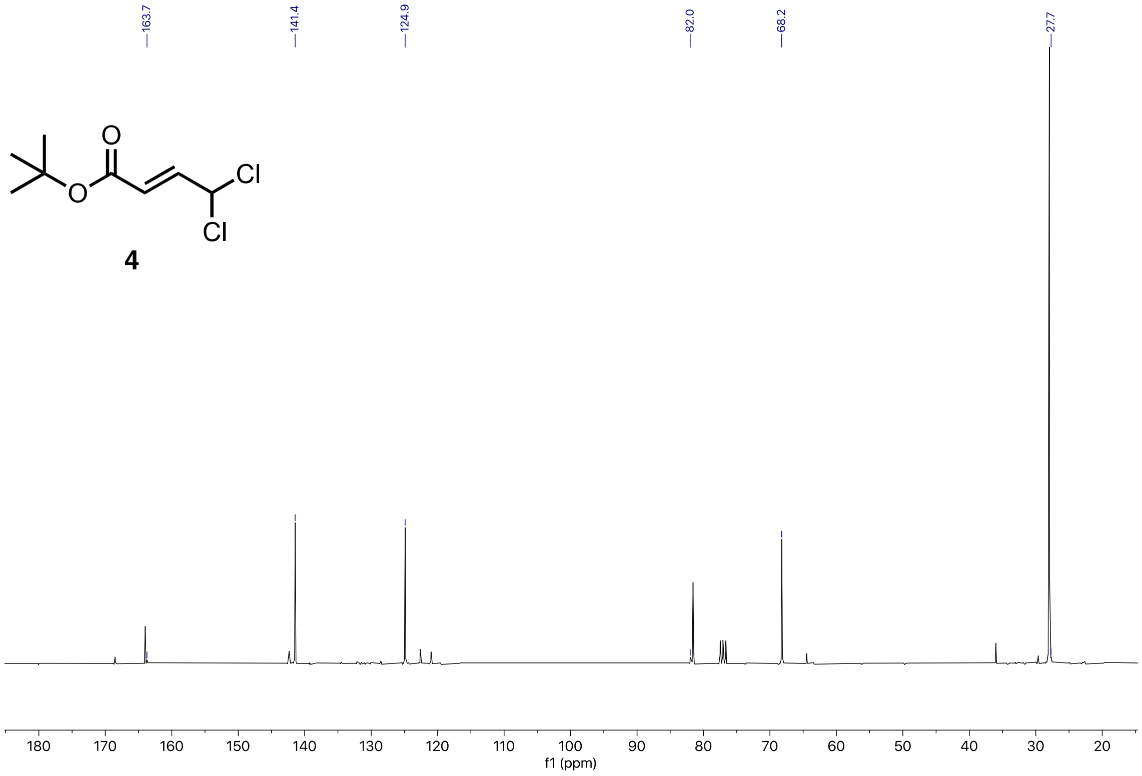

1H NMR (CDCl3, 300 MHz)

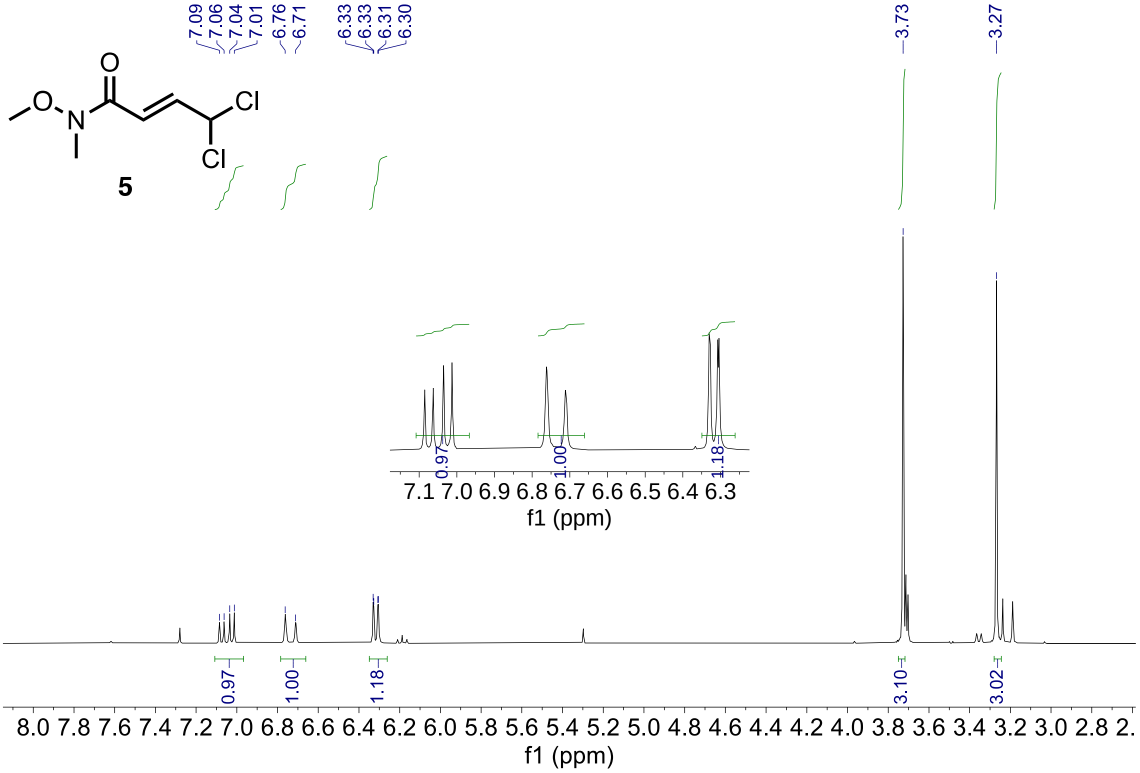

13C NMR (CDCl3, 300 MHz)}

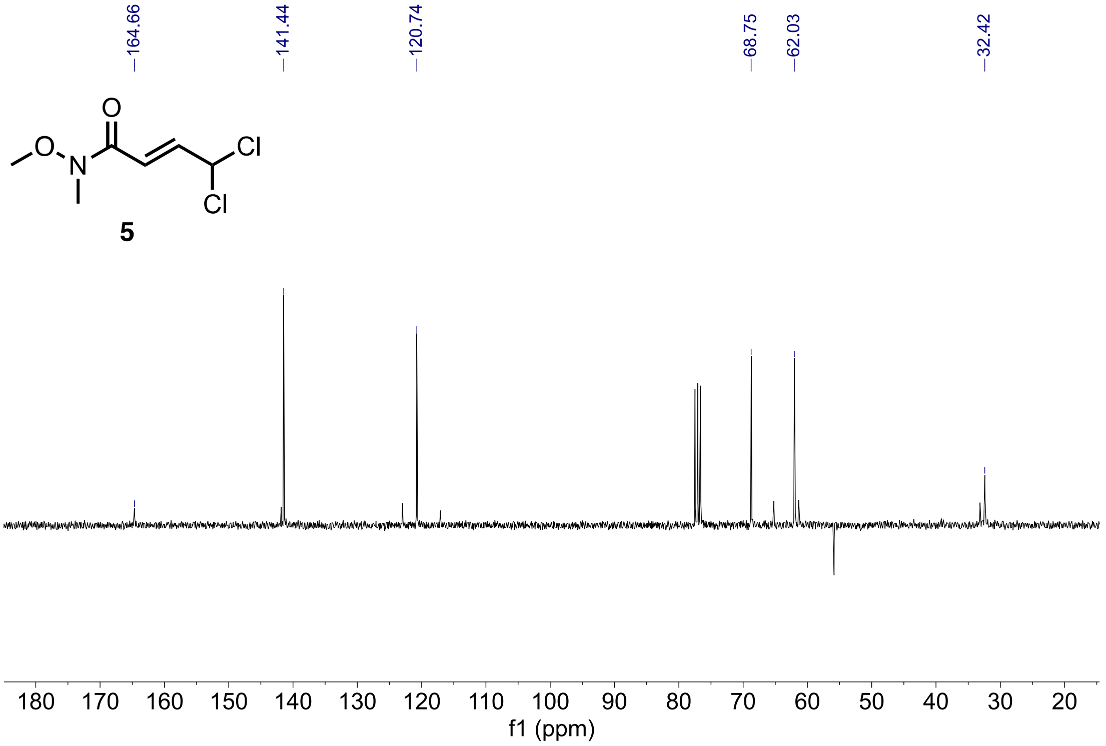

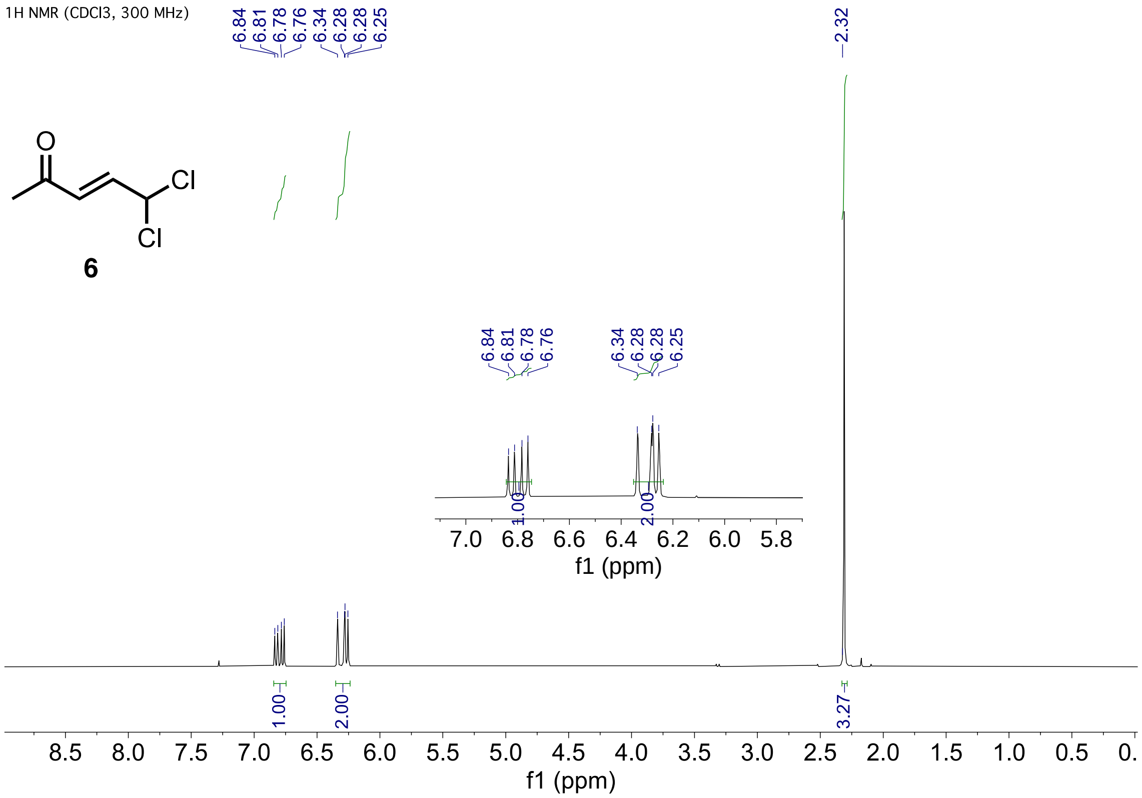
1H NMR (CDCl3, 300 MHz)
6.84
6.81
6.78
6.76
6.34
6.28
6.28
6.25
2.32
O
Cl
Cl
6
1.00
2.00
3.27
f1 (ppm)

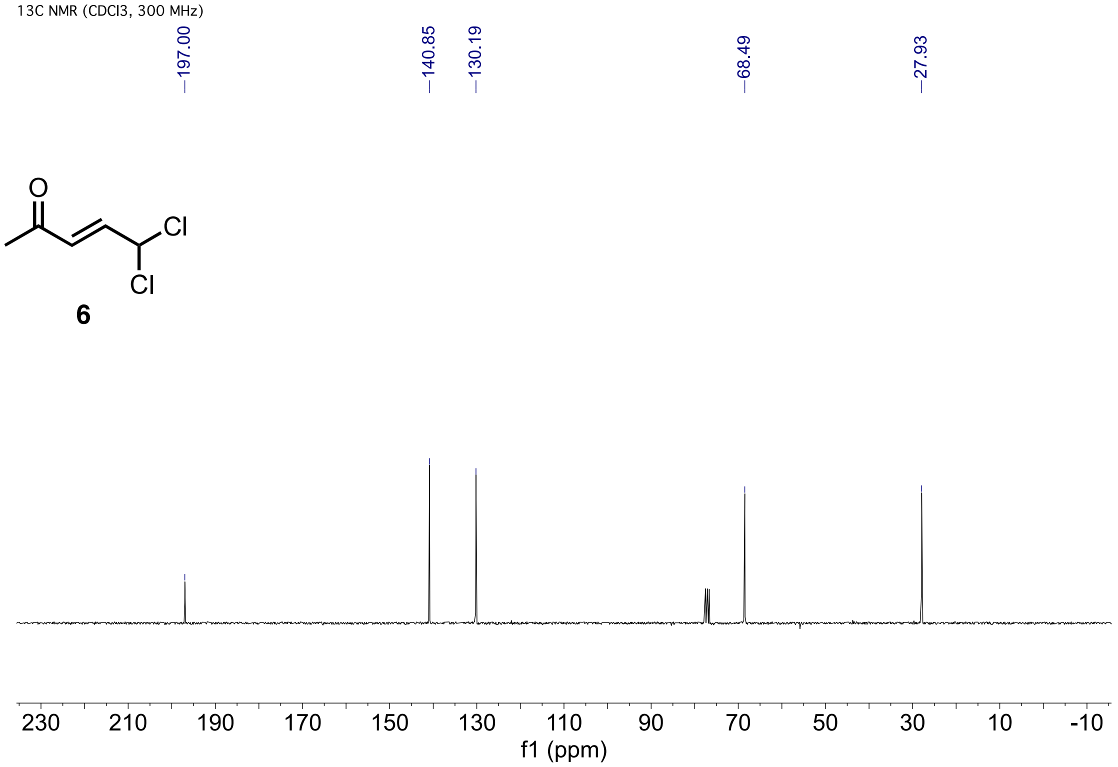
13C NMR (CDCl3, 300 MHz)
197.00
140.85
130.19
68.49
27.93
O
Cl
Cl
6
f1 (ppm)

1H NMR (CDCl3, 300 MHz)

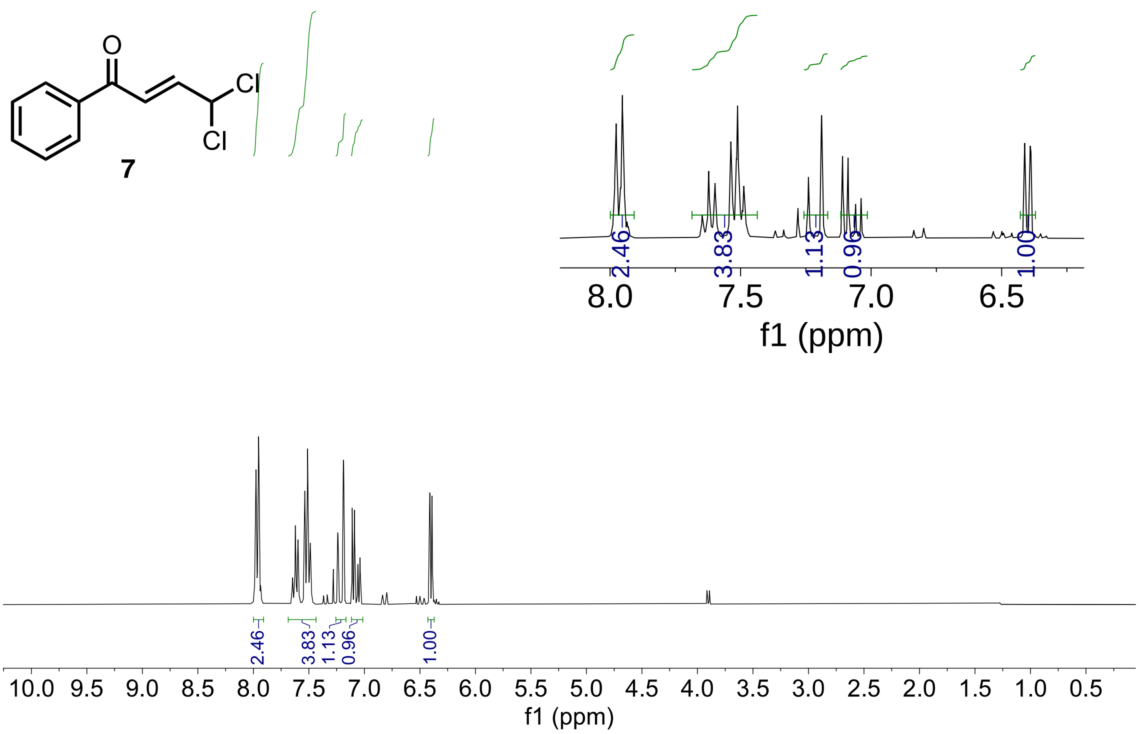

13C NMR (CDCl3, 300 MHz)

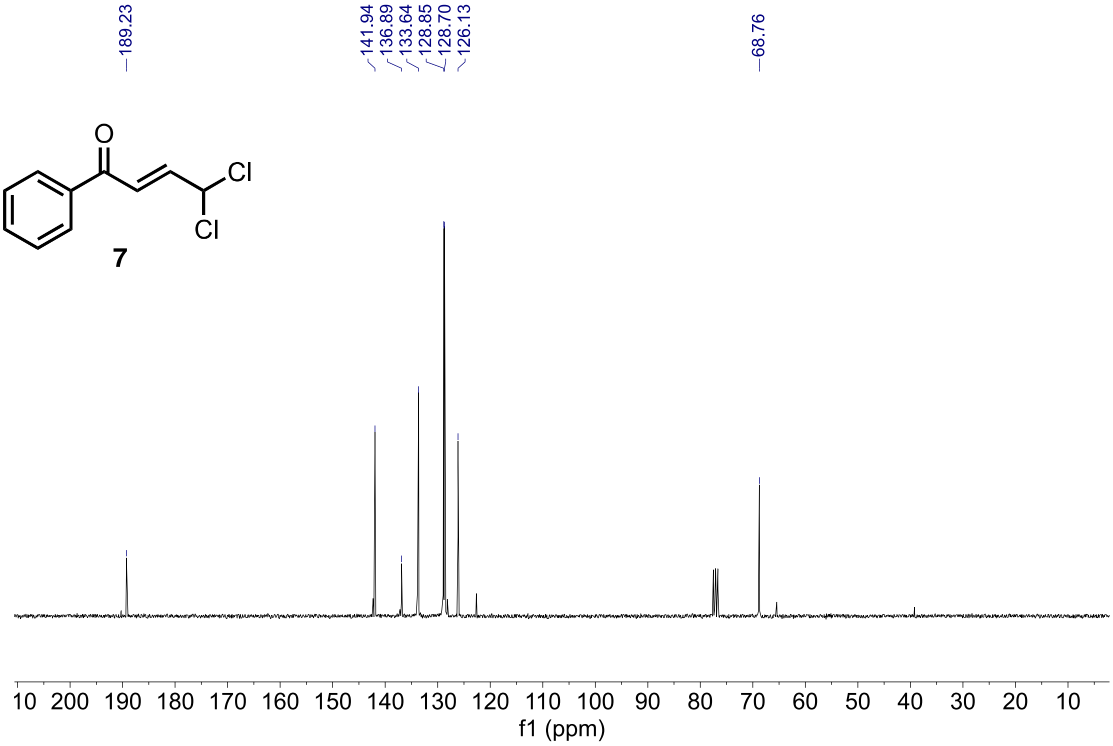

1H NMR (CDCl3, 500MHz)

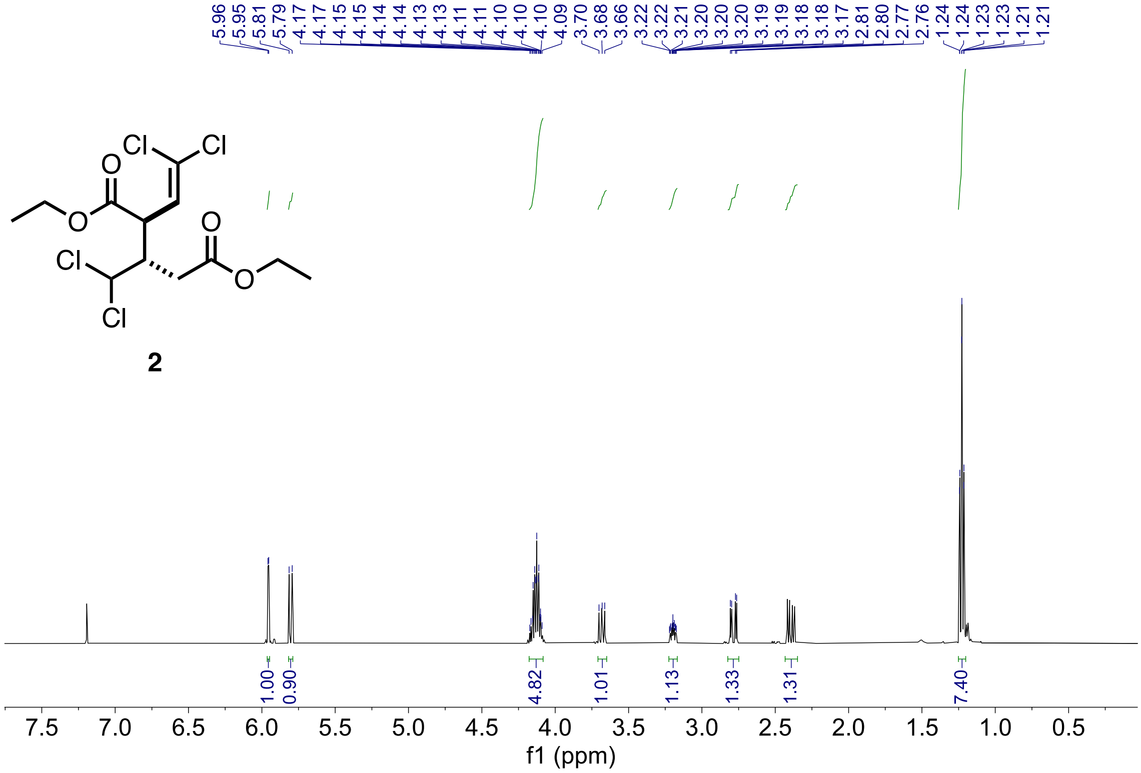

13C NMR (CDCl3, 500MHz)

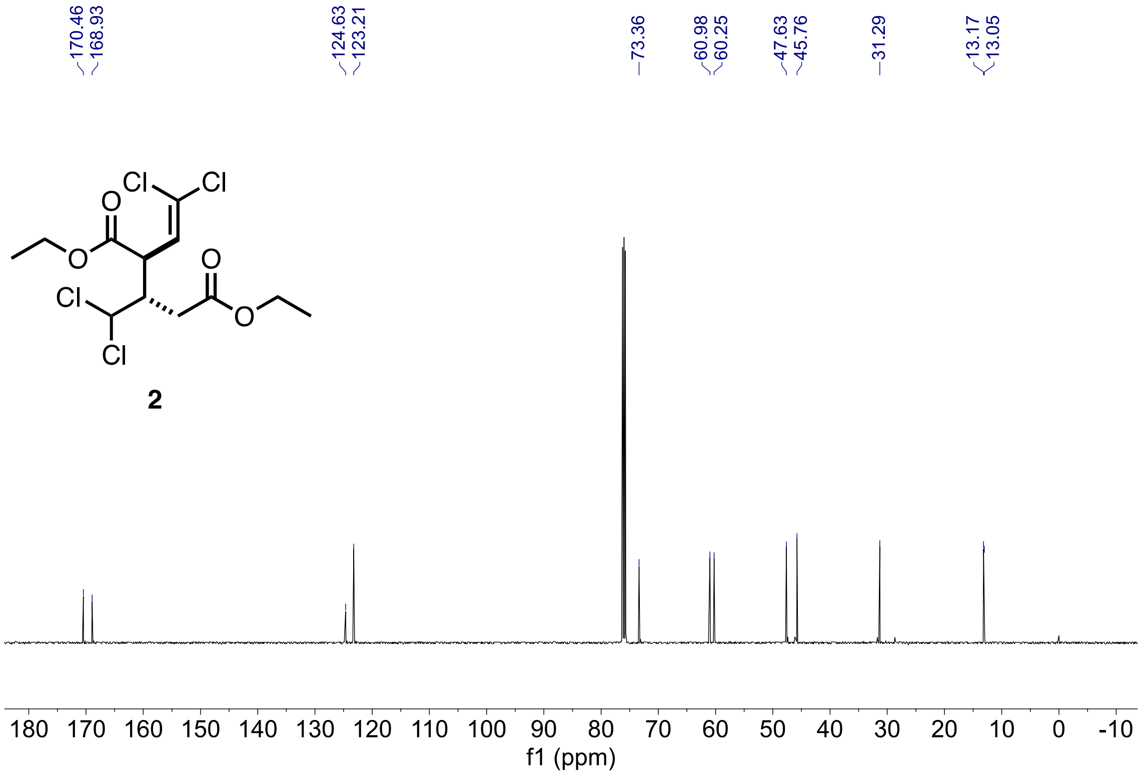

COSY

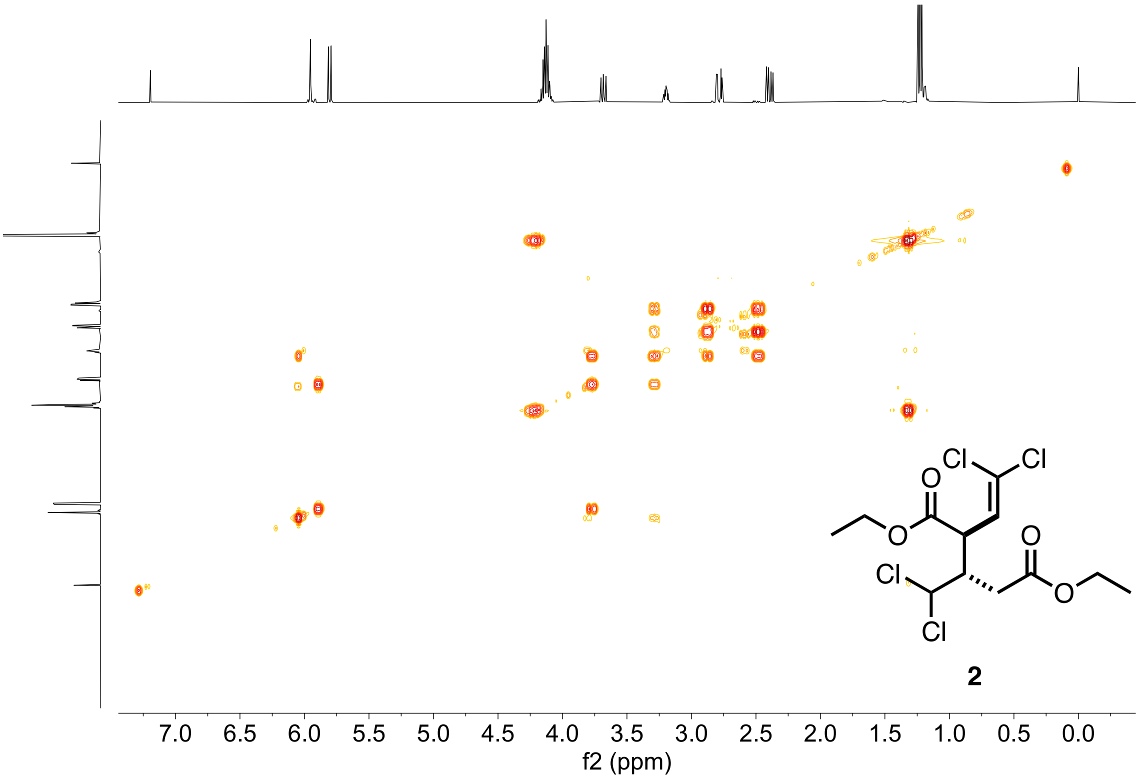
Cl
Cl
O
O
O
O
Cl
Cl
2
7.0
6.5
6.0
5.5
5.0
4.5
4.0
3.5
3.0
2.5
2.0
1.5
1.0
0.5
0.0
f2 (ppm)

HSQC

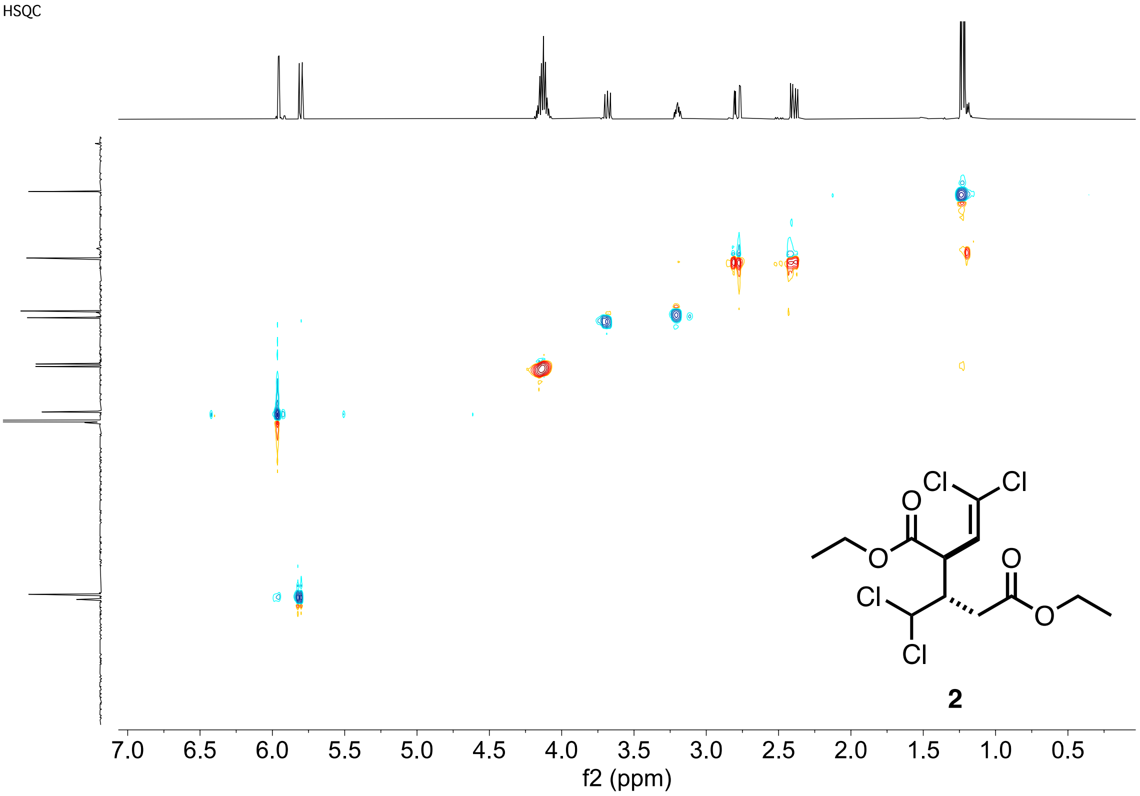
Cl
Cl
O
O
O
O
Cl
Cl
2
7.0
6.5
6.0
5.5
5.0
4.5
4.0
3.5
3.0
2.5
2.0
1.5
1.0
0.5
f2 (ppm)

HMBC

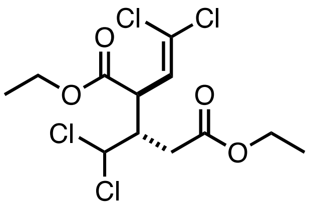
Cl
Cl
O
O
O
O
Cl
Cl

2

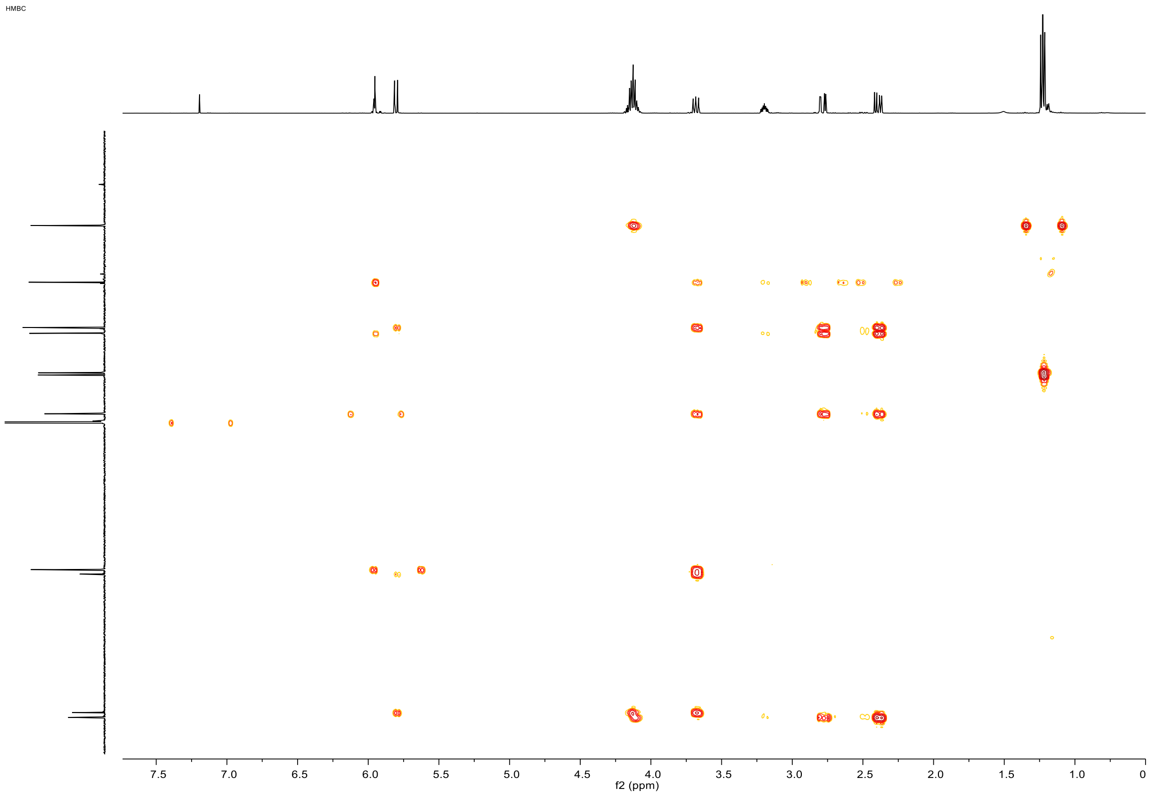
HMBC
7.5
7.0
6.5
6.0
5.5
5.0
4.5
4.0
3.5
3.0
2.5
2.0
1.5
1.0
0
f2 (ppm)

1H NMR (CDCl3, 300 MHz)

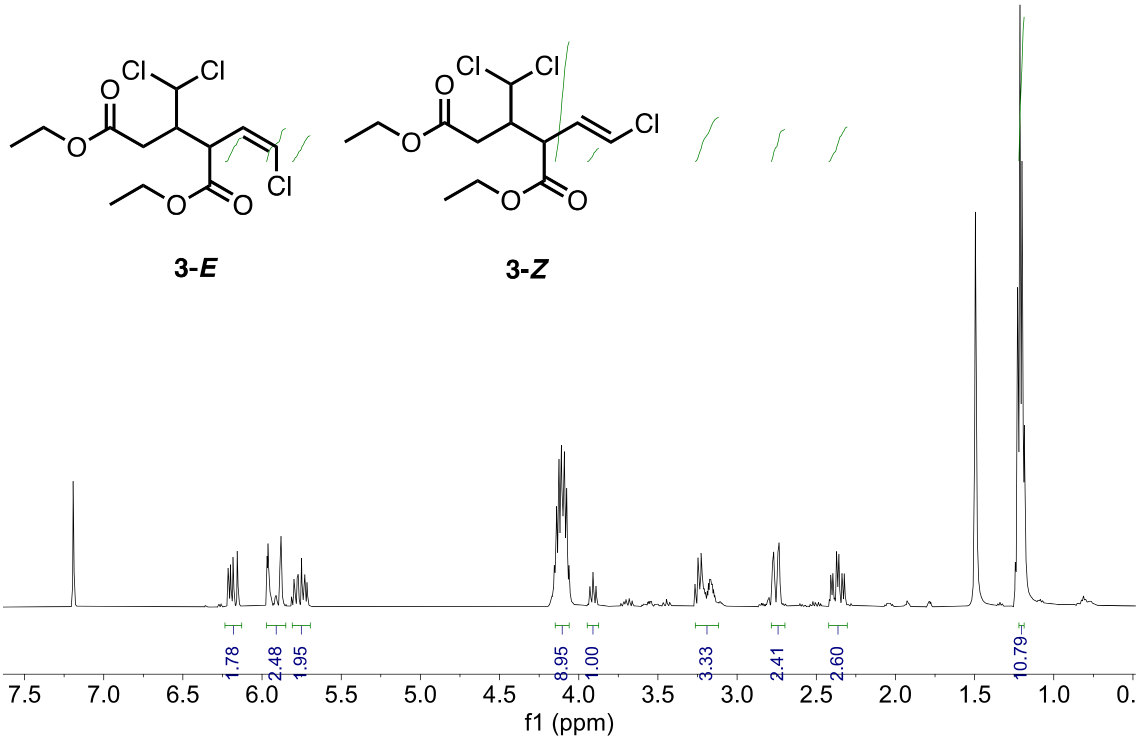

13C NMR (CDCl3, 500 MHz)

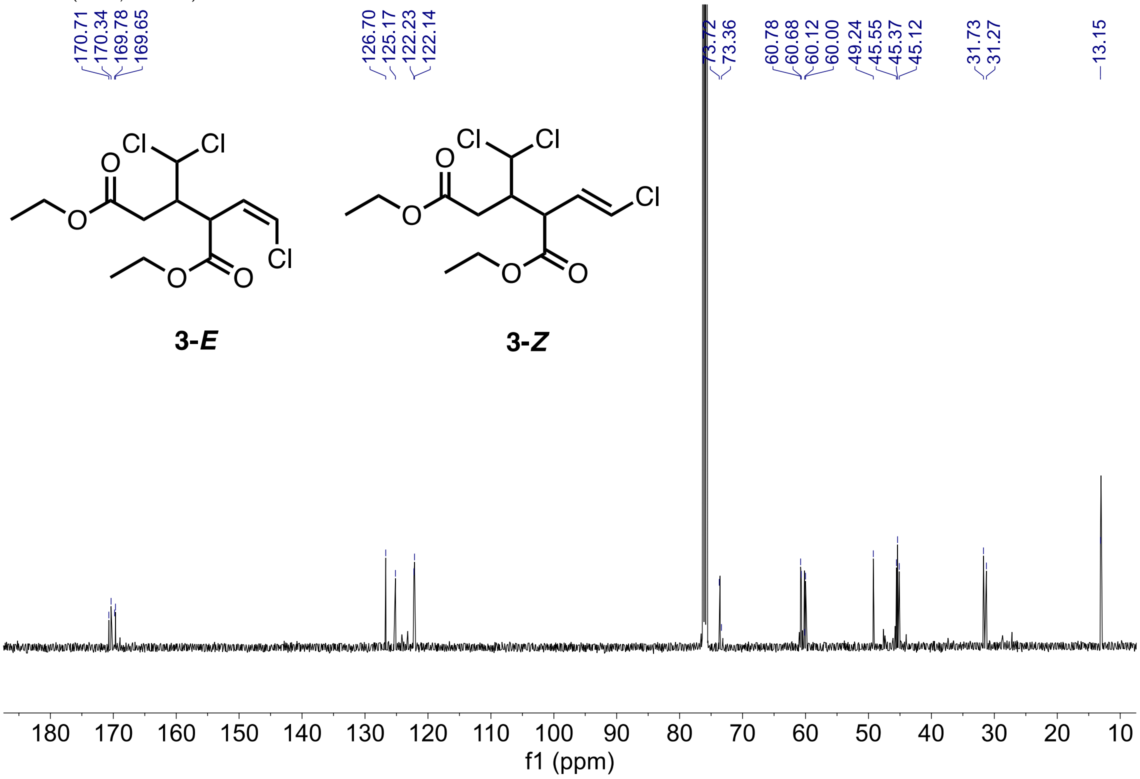

1H NMR (CDCl3, 300MHz)

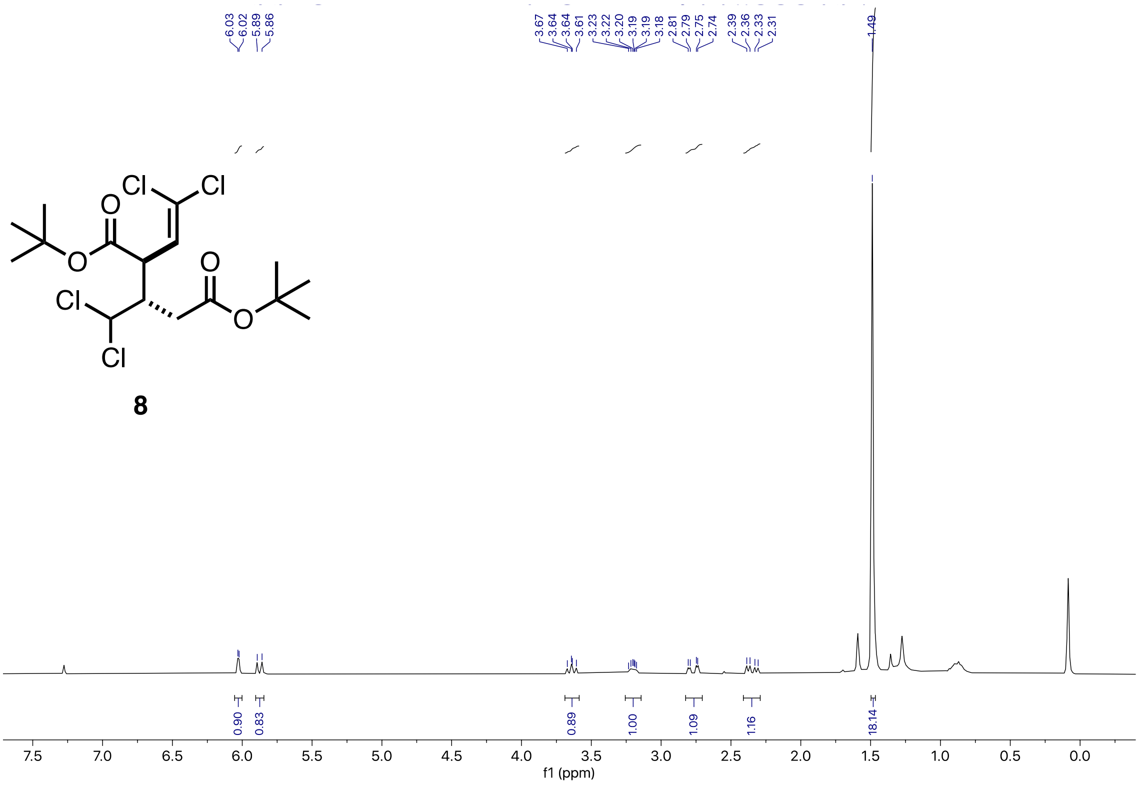

1H NMR (CDCl3, 300MHz)

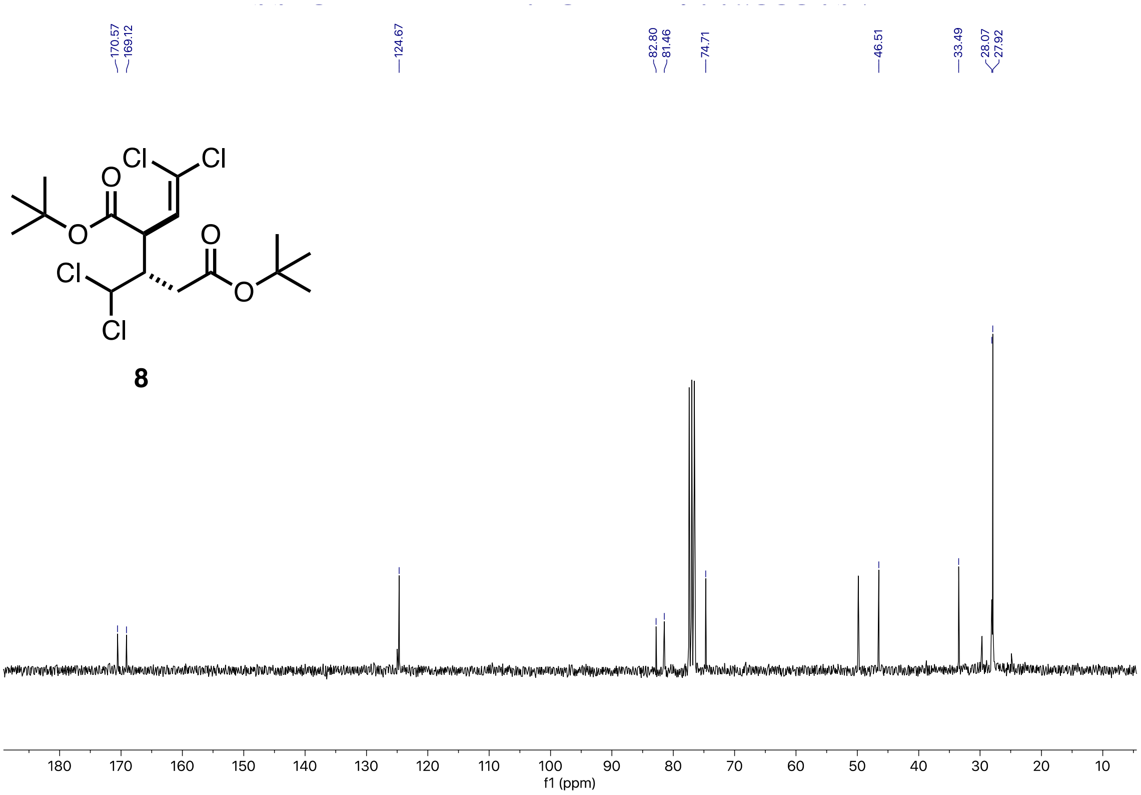

1H NMR (CDCl3, 300MHz)

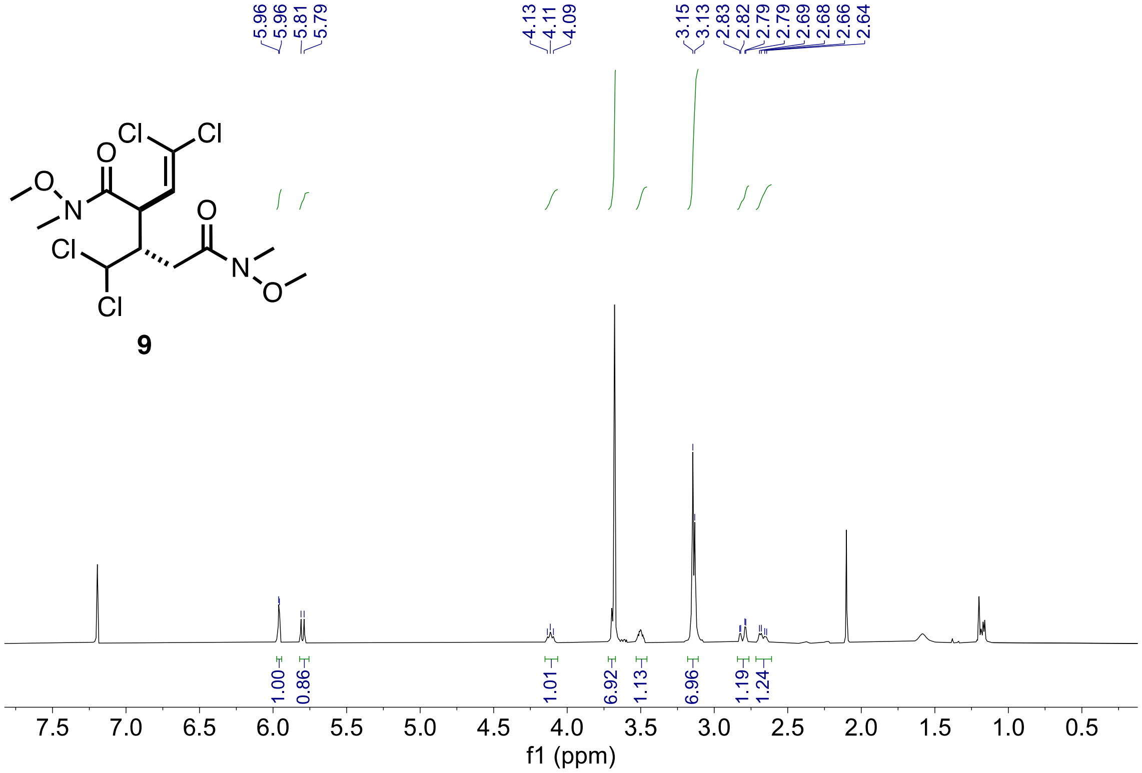

13C NMR (CDCl3, 500MHz)

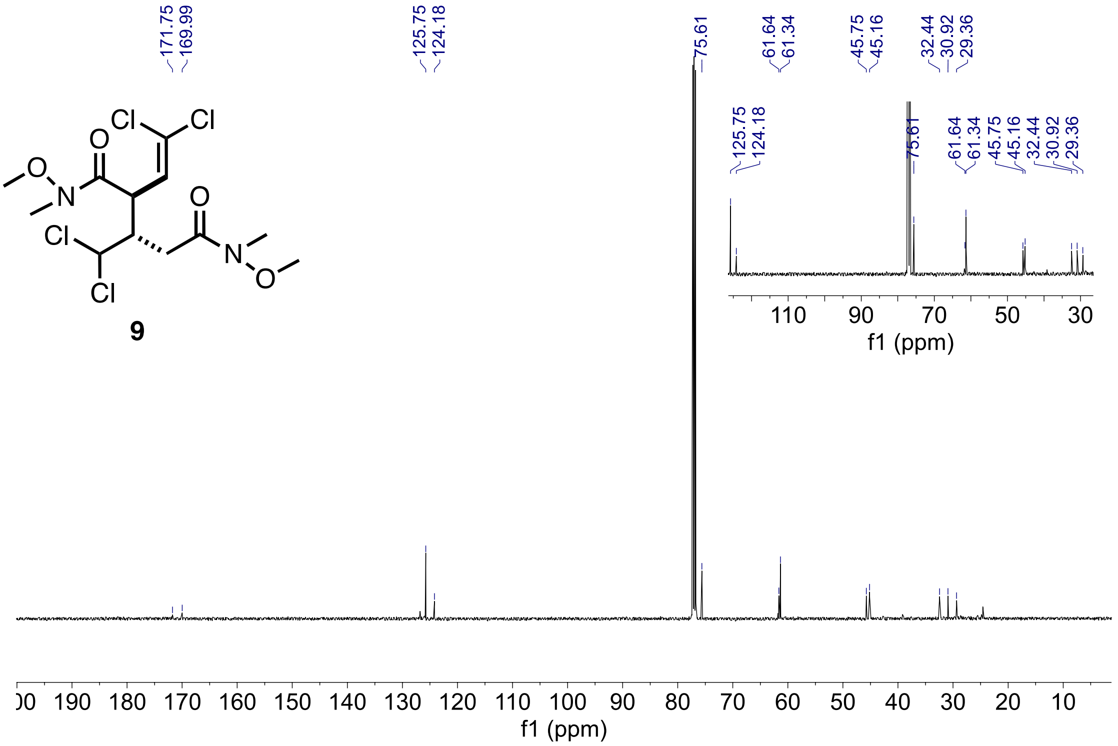

1H NMR (CDCl3, 500MHz)

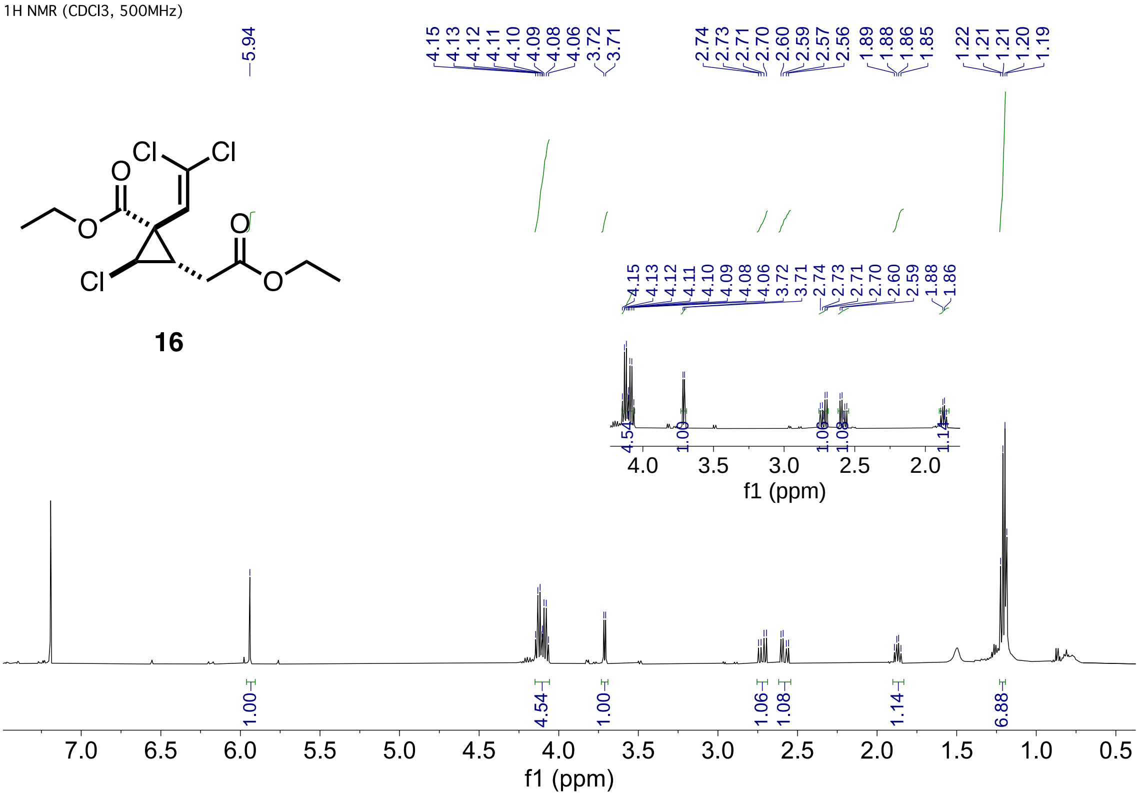

13C NMR (CDCl3, 500MHz)

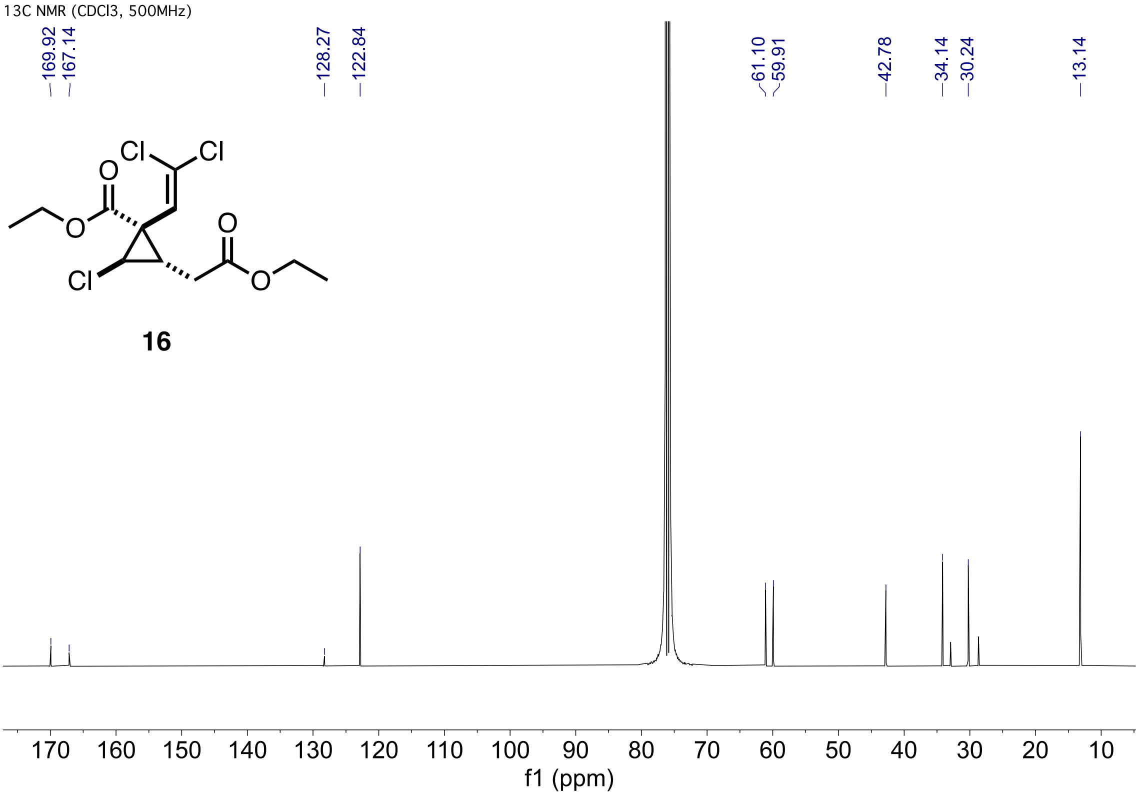

1H NMR (CDCl3, 300MHz)}

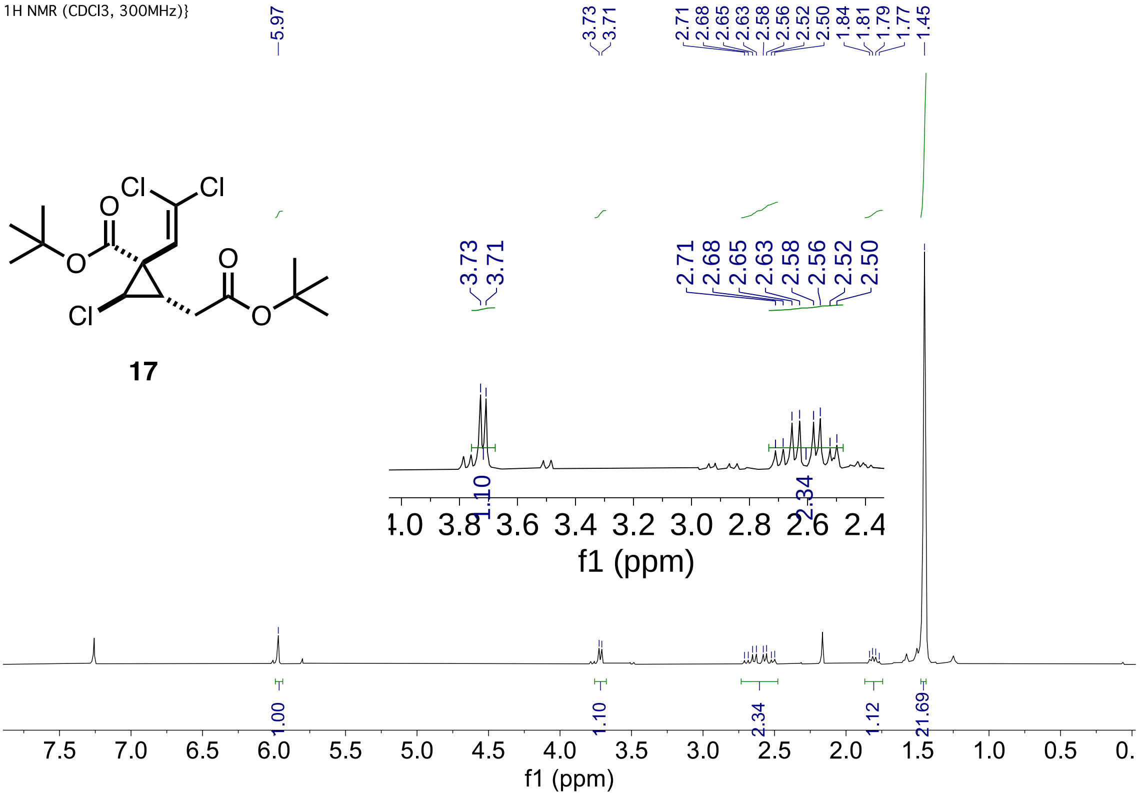

17

13C NMR (CDCl3, 300MHz)

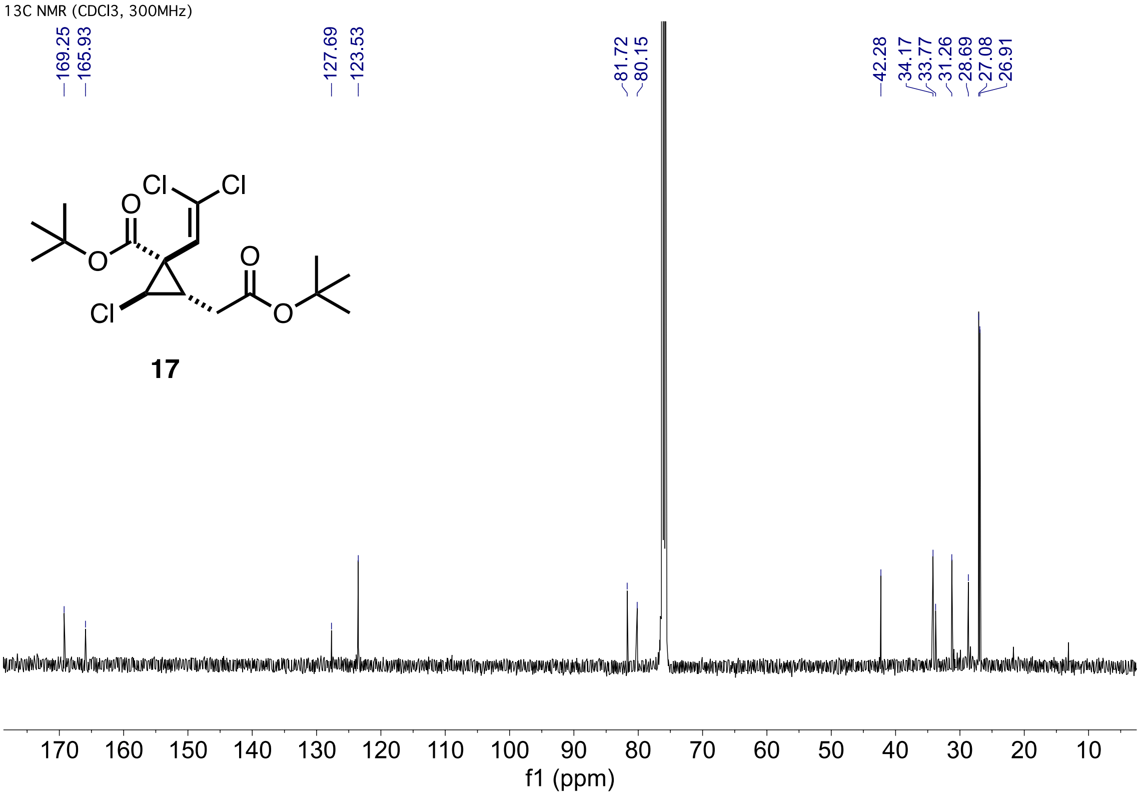

17

1H NMR (CDCl3, 300MHz)

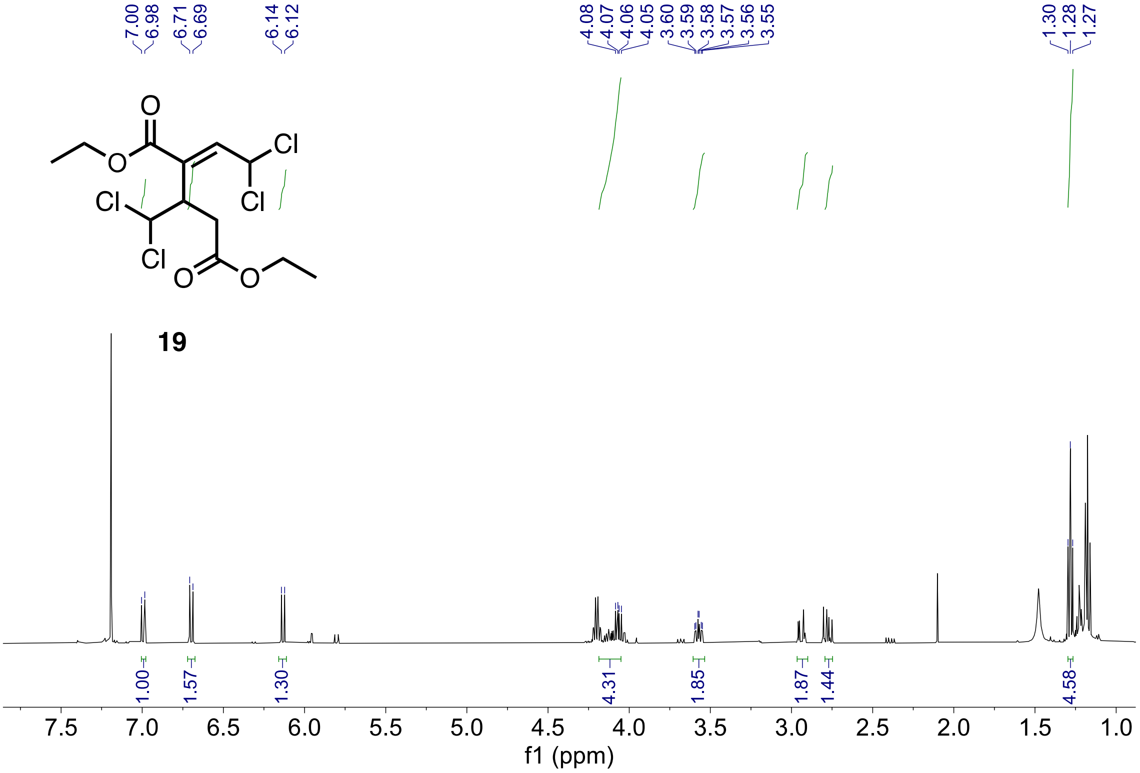

13C NMR (CDCl3, 300MHz)

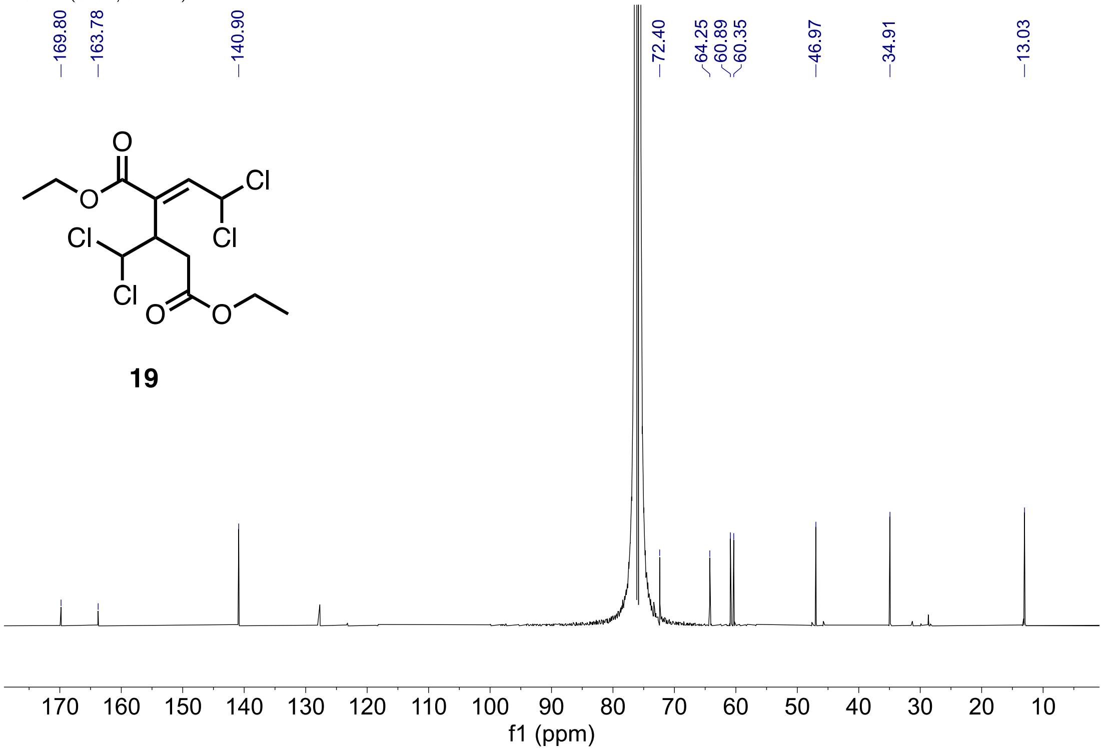

1H NMR (CDCl3, 300MHz)

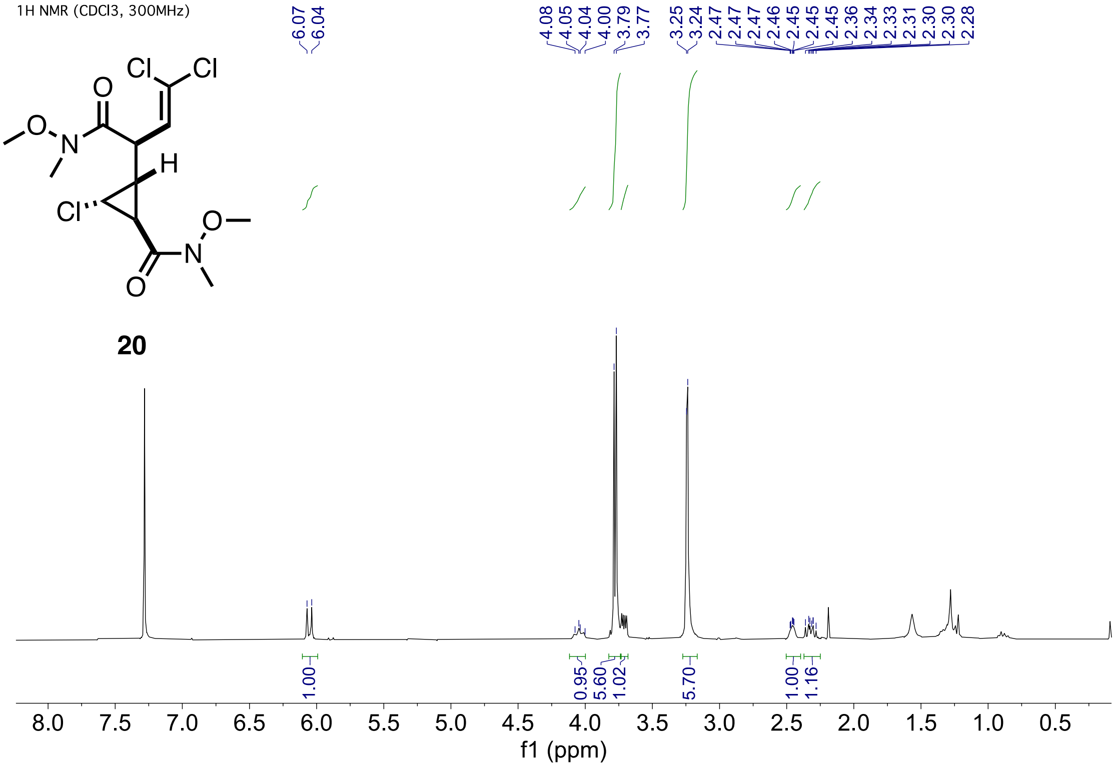

20

13C NMR (CDCl3, 500MHz)

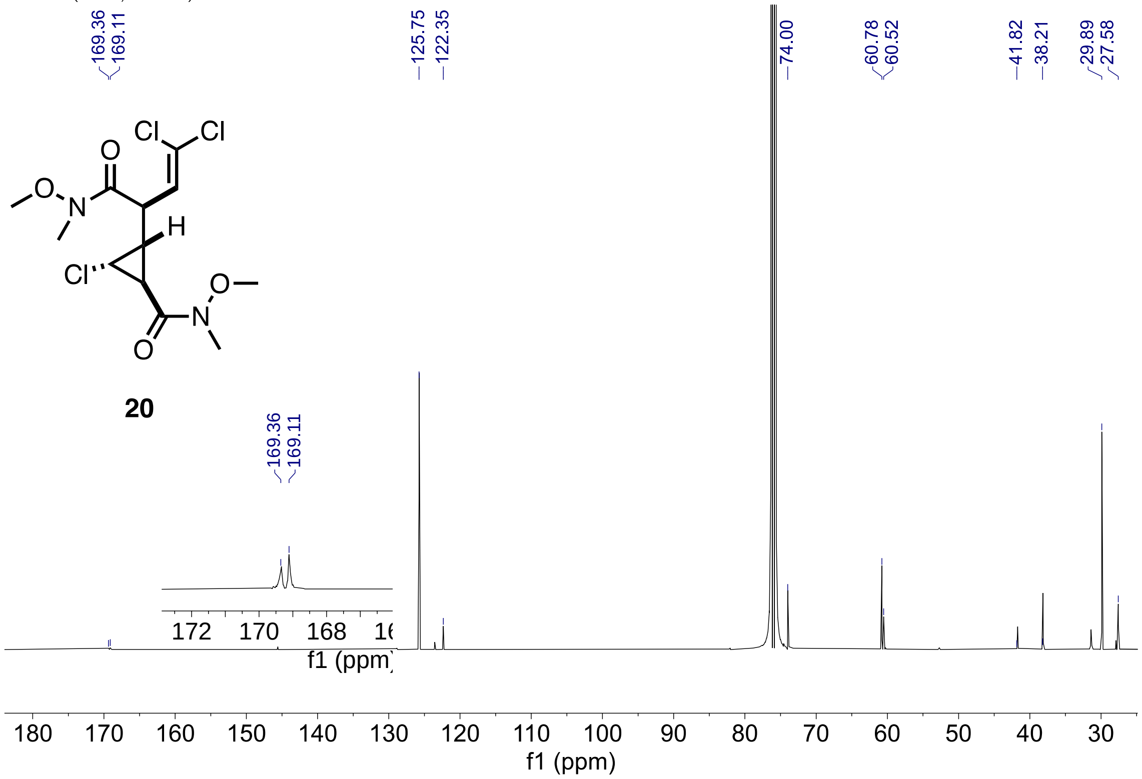

20

9. Stereochemical determination of products by NOESY experiment

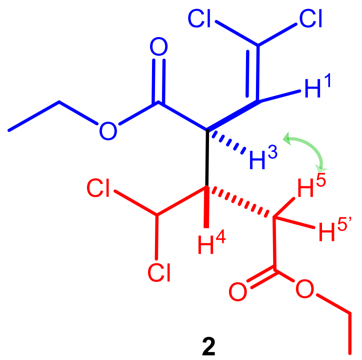

2

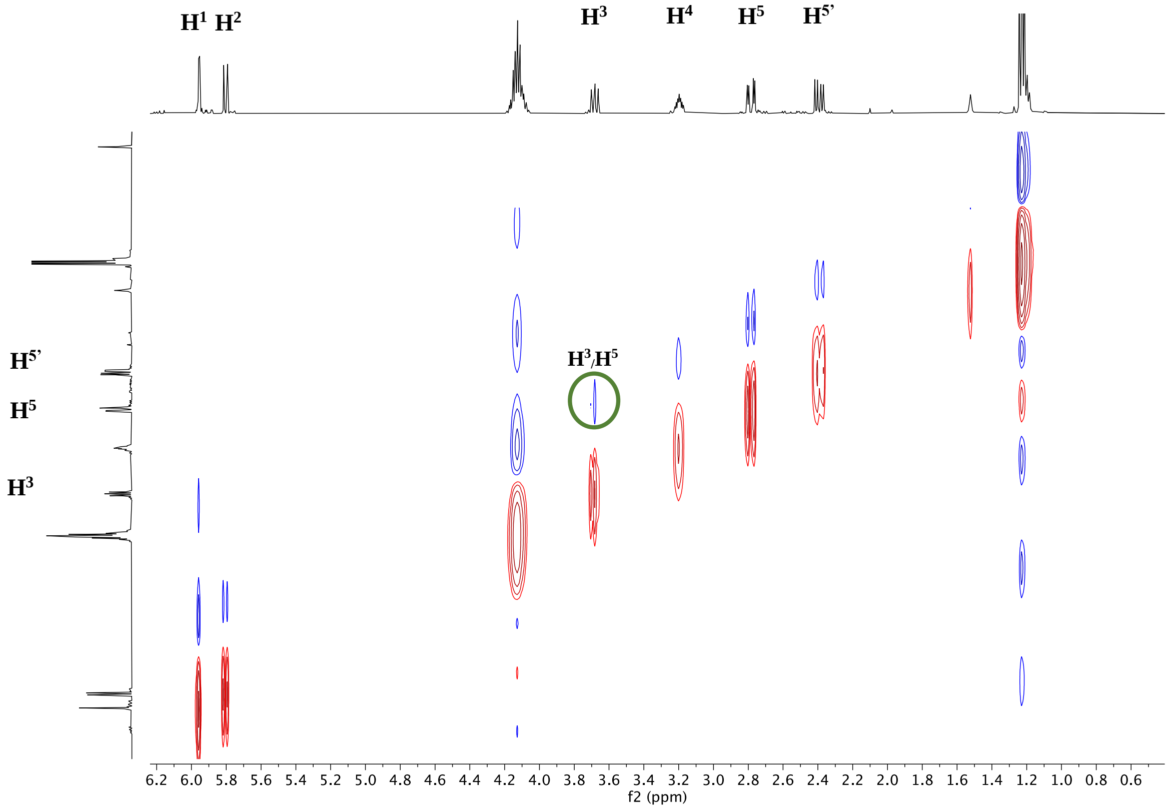

The cross peak between H^3/H^5 indicates that these protons are on the same side of the molecule. This supports the proposed stereochemistry.

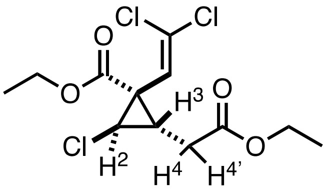

17

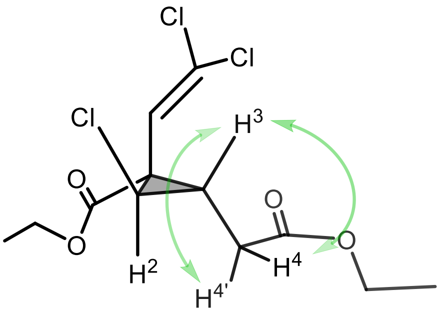

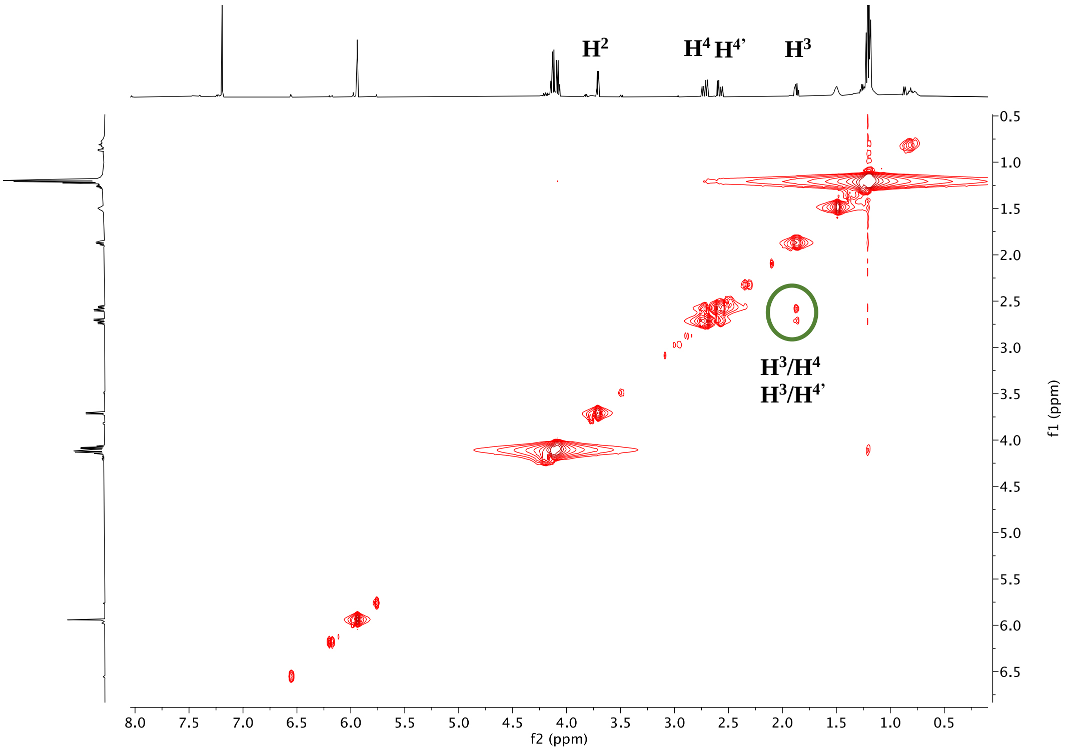

The single cross peak observed for cyclopropane protons H^3 and H^2 is the cross peak between H^3/H^5 (highlighted in green) due to the vicinal proximity of both carbons. The absence of cross peaks between H^3 and H^2 suggests that these protons are on opposite sides of the cyclopropane ring.

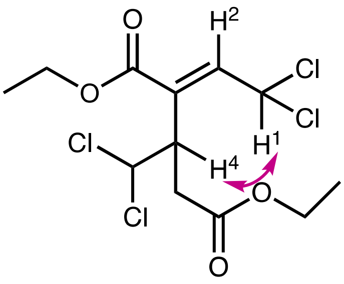

19

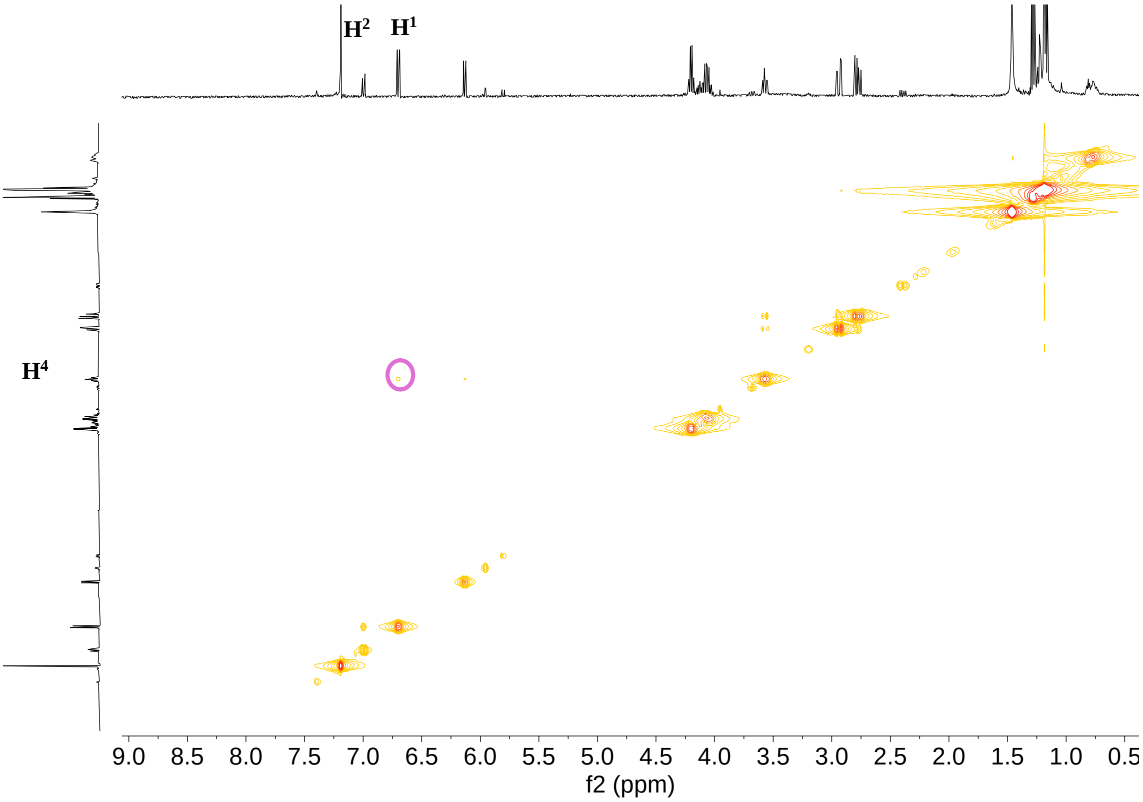

The cross peak between H^4/H^1 indicates that these protons are close in space so the configuration of the molecule is *E*.

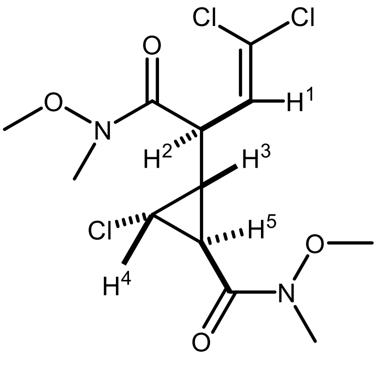

20

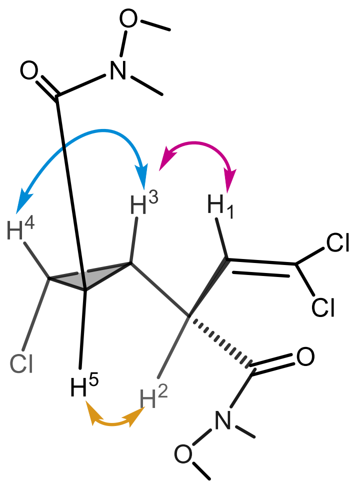

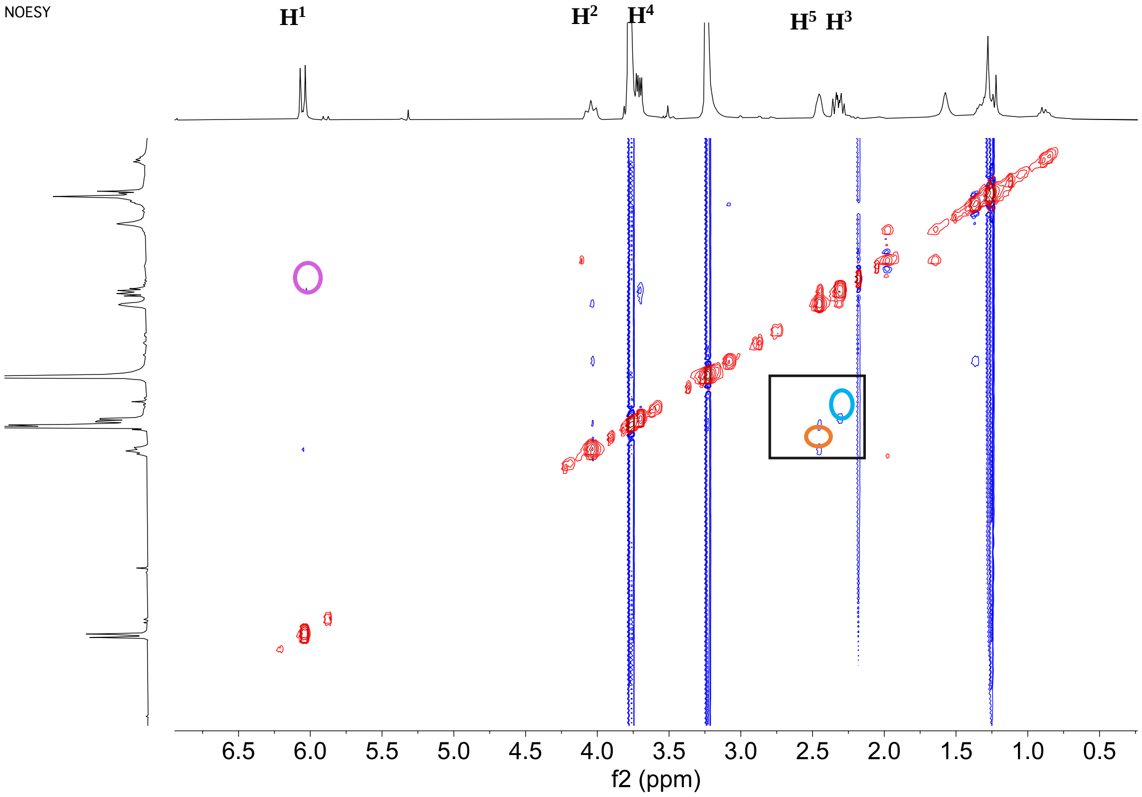

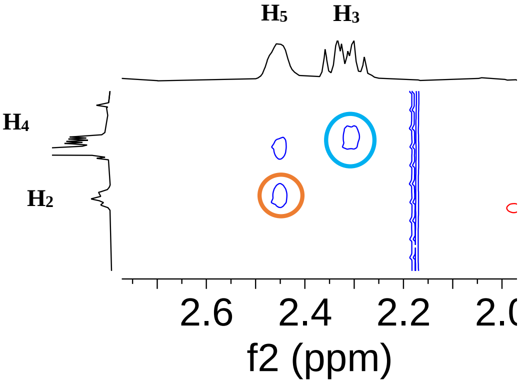

The cross peaks between H^3/H^4 (blue) and H^2/H^5 (orange) indicate that each pair of protons are on each side of the cyclopropane ring. Moreover, cross peaks between H^3/H^1 (pink) and H^2/H^5 (orange) support the relative configuration of the exocyclic stereocenter.
